# Perceiving depth from texture and disparity cues: Evidence for a non-probabilistic account of cue integration

**DOI:** 10.1167/jov.23.7.13

**Published:** 2023-07-24

**Authors:** Jovan T. Kemp, Evan Cesanek, Fulvio Domini

**Affiliations:** 1 Department of Cognitive, Linguistic, and Psychological Sciences, Brown University, Providence, RI, USA; 2 Mortimer B. Zuckerman Mind Brain Behavior Institute, Columbia University, New York, NY, USA; 3 Department of Cognitive, Linguistic, and Psychological Sciences, Brown University, Providence, RI, USA; 4 Italian Institute of Technology, Rovereto, Italy

**Keywords:** 3D vision, depth cue integration, shape from texture, shape from disparity, flatness cues

## Abstract

Bayesian inference theories have been extensively used to model how the brain derives three-dimensional (3D) information from ambiguous visual input. In particular, the maximum likelihood estimation (MLE) model combines estimates from multiple depth cues according to their relative reliability to produce the most probable 3D interpretation. Here, we tested an alternative theory of cue integration, termed the intrinsic constraint (IC) theory, which postulates that the visual system derives the most stable, not most probable, interpretation of the visual input amid variations in viewing conditions. The vector sum model provides a normative approach for achieving this goal where individual cue estimates are components of a multidimensional vector whose norm determines the combined estimate. Individual cue estimates are not accurate but related to distal 3D properties through a deterministic mapping. In three experiments, we show that the IC theory can more adeptly account for 3D cue integration than MLE models. In Experiment 1, we show systematic biases in the perception of depth from texture and depth from binocular disparity. Critically, we demonstrate that the vector sum model predicts an increase in perceived depth when these cues are combined. In Experiment 2, we illustrate the IC theory radical reinterpretation of the just noticeable difference (JND) and test the related vector sum model prediction of the classic finding of smaller JNDs for combined-cue versus single-cue stimuli. In Experiment 3, we confirm the vector sum prediction that biases found in cue integration experiments cannot be attributed to flatness cues, as the MLE model predicts.

## Introduction

A fundamental aspect of human visual perception is its ability to interpret three-dimensional (3D) space from patterns of light. We may be able to ignore color when judging brightness or divert our attention from specific objects with eye movements, but we cannot possibly suppress our experience of the 3D environment. The problem of how the visual system constructs a 3D interpretation from the two-dimensional (2D) manifold of light intensity on the retina has been approached during the last three decades through the Bayesian inference theory of 3D vision (Landy, Banks, & Knill, 2011; Landy, Maloney, Johnston, & Young, 1995). The intuitive appeal of this theory has led to a large number of empirical studies aimed at evaluating its predictions (Adams, Graf, & Ernst, 2004; Adams & Mamassian, 2004; Chen & Saunders, 2019; Ernst & Banks, 2002; Hillis, Ernst, Banks, & Landy, 2002; Hillis, Watt, Landy, & Banks, 2004; Jacobs, 1999; Jacobs, 2002; Knill, 1998a; Knill, 2007; Knill & Saunders, 2003; Mamassian & Landy, 1998; Saunders & Chen; 2015; Saunders & Knill, 2001; Schrater & Kersten, 2000; Welchman, Lam, & Bulthoff, 2008; Young, Landy, & Maloney, 1993). Although this approach successfully accounts for a wide range of findings, it is unable to predict many fundamental real-world phenomena, such as systematic biases in 3D judgments (Bozzacchi & Domini, 2015; Bozzacchi, Volcic, & Domini, 2016; Campagnoli, Croom, & Domini, 2017; Caudek & Domini, 1998; Domini & Braunstein, 1998; Domini & Caudek, 1999; Domini & Caudek, 2003; Domini, Caudek, & Richman, 1998; Egan & Todd, 2015; Fantoni, Caudek, & Domini, 2010; Kopiske, Bozzacchi, Volcic, & Domini, 2019; Liu & Todd, 2004; Norman, Todd, & Orban, 2004; Norman, Todd, Perotti, & Tittle, 1996; Norman, Todd, & Phillips, 1995; Perotti, Todd, Lappin, & Phillips, 1998; Phillips & Todd, 1996; Tittle, Todd, Perotti, & Norman, 1995; Todd, 2004; Todd & Bressan, 1990; Todd, Chen, & Norman, 1998; Todd, Egan, & Phillips, 2014; Todd & Norman, 2003; Todd & Thaler, 2010; Todd, Thaler, & Dijkstra, 2005; Todd, Thaler, Dijkstra, Koenderink, & Kappers, 2007; Todd, Tittle, & Norman, 1995; Volcic, Fantoni, Caudek, Assad, & Domini, 2013), internal inconsistencies among judgments at different scales (Lappin & Craft, 2000; Loomis, Da Silva, Philbeck, & Fukusima, 1996; Loomis, Philbeck, & Zahorik, 2002), the paradox of pictorial depth and pictorial duality (Haber, 1980; Koenderink, 1998; Koenderink, van Doorn, Kappers, & Todd, 2001; Vishwanath, 2011; Vishwanath, 2013; Vishwanath, 2014; Vishwanath, 2020), and differences in phenomenology of 3D vision (Koenderink, van Doorn, & Wagemans, 2015; Koenderink, van Doorn, & Wagemans, 2018; Vishwanath, 2013). In this paper, we test a new theoretical framework based on an entirely different set of assumptions that can more parsimoniously account for a large range of observations in 3D perception.

There are two main assumptions that have guided recent research in 3D vision: (1) Independent modules derive noisy estimates that are on average veridical (i.e., unbiased) (Clark & Yuille, 1990; Landy et al., 2011) and (2) visual mechanisms estimate the level of sensory noise, such that the outputs of individual modules represent probability distributions. Representing probability distributions enables the statistically optimal combination of independent estimates, as proposed by Bayesian integration frameworks (e.g., Landy et al., 2011). Although there are more general implementations of Bayesian combinations, in this paper we focus on the linear maximum likelihood estimation (MLE) model (Ernst & Bülthoff, 2004), following similar past studies that have assumed a negligible influence of priors when viewing objects defined by binocular disparity, texture, or both (Chen & Saunders, 2020; Hillis et al., 2004; Johnston, Cumming, & Parker, 1993; Knill & Saunders, 2003).

The predictions of the linear MLE model for the integration of texture and disparity information can be summarized by two equations. First, if ${\hat{z}}_{t}$ and ${\hat{z}}_{d}$ are the depth estimates from the texture and disparity modules and σ*_t_* and σ*_d_* are the standard deviations of the noise of these estimates, then the combined estimate ${\hat{z}}_{c}$ is a weighted average with weights proportional to the reliabilities, $r_{i}=\frac{1}{\sigma_{i}^{2}}$, of the estimates:

(1) $$\begin{array}{c} {\hat{z}}_{c}=w_{t}{\hat{z}}_{t}+w_{d}{\hat{z}}_{d}\; \end{array}$$

where $w_{t}=\frac{r_{t}}{r_{d}+r_{t}}$ and $w_{d}=\frac{r_{d}}{r_{d}+r_{t}}$. Second, the variance of the combined estimate is smaller than that of either single-cue estimate, as predicted by the following relationship:

(2) $$\begin{array}{c} \sigma_{c}^{2}=\frac{\sigma_{d}^{2}\sigma_{t}^{2}}{\sigma_{d}^{2}+\sigma_{t}^{2}}=\frac{1}{\frac{1}{\sigma_{d}^{2}}+\frac{1}{\sigma_{t}^{2}}}.\; \end{array}$$

Although applying the MLE model to explain perceptual processing may appear straightforward, some of its core assumptions seem not to be satisfied by human perceptual systems. First, many experiments have shown that depth cues generally fail to produce accurate percepts, contrary to the veridicality assumption (Bozzacchi & Domini, 2015; Bozzacchi et al., 2016; Campagnoli et al., 2017; Caudek & Domini, 1998; Domini & Braunstein, 1998; Domini & Caudek, 1999; Domini & Caudek, 2003; Domini et al., 1998; Egan & Todd, 2015; Fantoni et al., 2010; Kopiske et al., 2019; Liu & Todd, 2004; Norman et al., 1996; Norman et al., 1995; Norman et al., 2004; Perotti et al., 1998; Phillips & Todd, 1996; Tittle et al., 1995; Todd, 2004; Todd & Bressan, 1990; Todd & Norman, 2003; Todd & Thaler, 2010; Todd et al., 1995; Todd et al., 1998; Todd et al., 2005; Todd et al., 2007; Todd et al., 2014; Volcic et al., 2013).

Second, it has been shown that, when perception is measured with techniques other than depth discrimination (e.g., by setting an independent 2D probe), the measured variability in perceived depth does not predict the relative weighting of depth cues (Todd, Christensen, & Guckes, 2010), contrary to the assumption that cue estimates are represented as probability distributions. These considerations suggest that the widespread application of the MLE model to human 3D perception may be inappropriate and that cue combination experiments need to be reinterpreted with an alternative explanation.

Here, we aim to describe a theoretical framework of 3D cue combinations that does not require the controversial assumptions of the mainstream MLE account described above. Instead, this framework assumes a derivation of 3D property estimates that (1) are generally biased but under some viewing conditions may be veridical, and (2) are deterministic rather than probabilistic. Variance within a cue estimate is negligible and therefore does not factor into the combination rule for multiple signals. Instead, this process is optimized to achieve perceptual stability in the face of natural variations of viewing conditions and surface properties (e.g., surface texture, reflectance functions). In the next section, we provide a formal specification of this framework that makes specific quantitative predictions. We then test these predictions in three experiments. Notably, we find that the model accurately predicts the increase in perceived depth and the reduction in discrimination threshold that occur when additional depth cues are added to a stimulus. This latter finding has been interpreted as a critical piece of evidence for the MLE model, but here we show that it is entirely consistent with our novel framework. Moreover, our model predicts several novel results that cannot be predicted by previous inference theories of cue integration.

## Intrinsic constraint theory of multi-cue processing

The normative model we propose is termed the intrinsic constraint (IC) theory, in reference to the original model from which it was developed (Domini & Caudek, 2009; Domini, Caudek, & Tassinari, 2006). However, the model we propose here is based on entirely different assumptions than the earlier IC theory.

**Figure 1.**
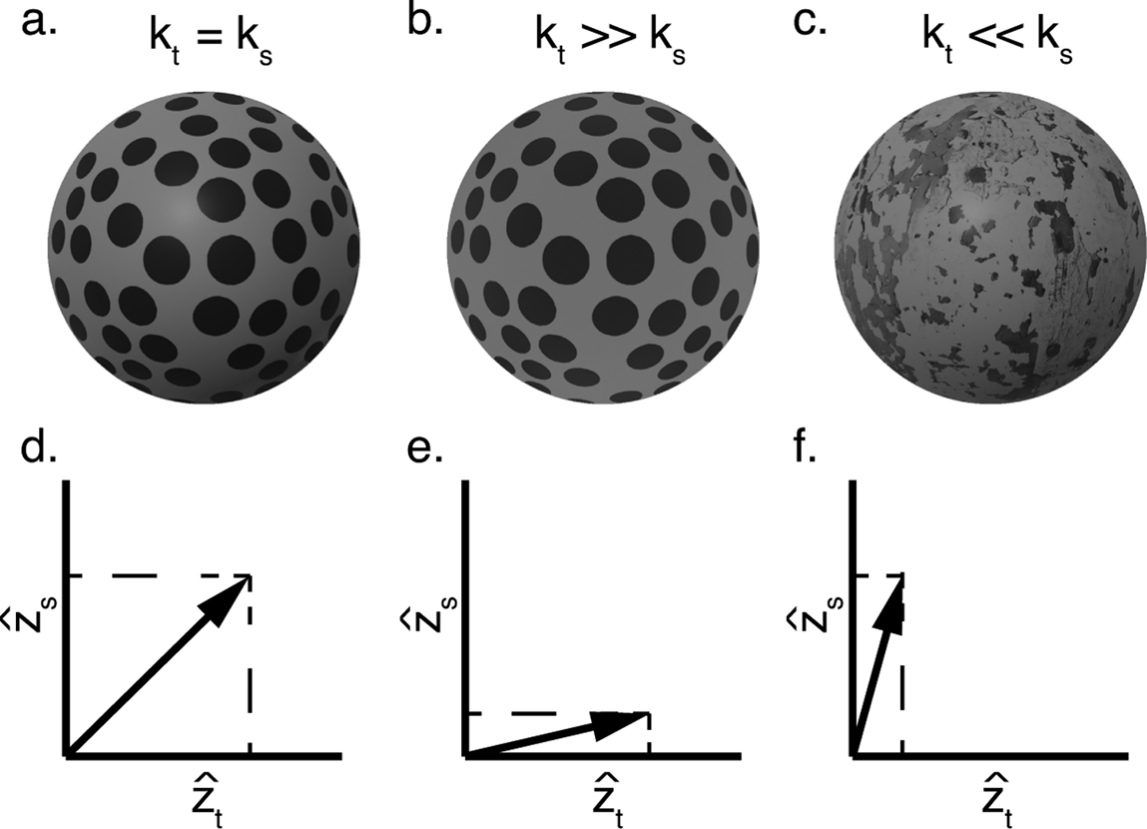
(a–c) A series of rounded surfaces with different levels of texture and shading gradients. The cue strength, *k_i_*, depends on the “quality” of the gradients, with more detectible gradients producing larger cue strengths. (a) Textures with identifiable, regular elements and shading gradients with continuously varying luminance intensities produce large cue strengths that in this example are assumed to be identical. (b) Shading with small luminance differences and (c) textures with ambiguous elements produce smaller cue strengths. (d) Each cue is analyzed by an independent function that produces a depth signal, which is linearly proportional to the distal depth. These signals exist in a multidimensional space where the vector length of the signals depends on the depth of the surface and the cue strength of the cue. It is this vector length that drives perceived depth magnitude. When both cues form large gradients, then the vector length and subsequent perceived depth will be large. (e, f) Smaller gradients for either cue will reduce this vector length and the perceived depth.

First, we postulate that separate visual modules independently process distinct image regularities. We assume these modules are tuned to approximate a linear mapping between the distal 3D property and the internal 3D estimate ${\hat{z}}_{i}$, of the form ${\hat{z}}_{i}=k_{i}z$. The slope, *k_i_*, of this linear function, which we term the perceptual function, depends on the quality of the visual information within cue *i*. For example, from the images in Figures 1a to 1c, a texture module extracts the systematic change in shape and spatial frequency of texture elements resulting in an estimate$\;{\hat{z}}_{t}=k_{t}z$. Critically, in direct contrast to the MLE model, there is no assumption that the perceptual function is veridical, nor is there any explicit representation of the associated sensory noise. Therefore, removing the veridicality assumption makes the IC theory far more parsimonious than the MLE model.

The IC theory defines visual modules as independent insofar as the slopes, *k*, of the perceptual functions vary independently from one another due to influences from variables unrelated to 3D shape. Although for simplicity we refer to the module outputs as depth estimates, formally they are modeled as producing orthogonal signals in a multidimensional space where the vector magnitude of these signals indicates the magnitude of perceived depth. To illustrate the independence of these functions and their relationship in the multidimensional space, consider the surface depicted in Figure 1a. Along with the image signals emerging from the projection of texture elements, there is a shading gradient that also induces an independent image signal related to depth. However, the surface may be viewed in a different lighting condition. For example, overcast weather would wash out the shading gradient while leaving the texture pattern unmodified, resulting in the image shown in Figure 1b. On the other hand, the same surface can be covered with irregular texture rather than the highly regular polka dots while the lighting condition remains identical to Figure 1a. The shading pattern still produces a smooth luminance gradient but there would be a less clear gradient of texture element deformation, as depicted in Figure 1c. In these examples, independent nuisance variables are associated with the surface properties and the sources of illumination. In a similar fashion, different nuisance variables will affect other image signals, such as the speed of the observer in motion parallax (Fantoni, Caudek, & Domini, 2012) or the fixation distance between the observer and the object in binocular disparities (Johnston, 1991). In general, nuisance variables describe factors that influence the quality of depth cues and therefore cause changes in perceived depth while the distal 3D surface remains constant. In other words, despite being independent of the surface shape, nuisance variables influence the slopes of the perceptual functions.

Because the slope of the perceptual functions, *k*, indicates the response to distal depth from individual cues,^1^ we refer to these parameters as *cue strengths*. The independence of cue strengths allows us to represent the totality of the cue estimates derived from a given stimulus as a multidimensional vector with each orthogonal axes representing the information from each cue. This is illustrated in Figures 1d to 1f, which show the specific examples of 2D vectors representing texture and shading information contained in the stimuli of Figures 1a to 1c. Figure 1d represents the depth information carried by the stimulus in Figure 1a, for which texture and shading are assumed (for illustration purposes) to have the same strength (*k_t_* = *k_s_*). In Figure 1e, the strength of texture is much greater than the strength of shading, as the shading has been removed (*k_t_* > *k_s_*). In Figure 1f, the strength of shading is greater than the strength of texture, as the texture is highly irregular (*k_t_* < *k_s_*). Because both ${\hat{z}}_{t}$ and ${\hat{z}}_{s}\;$are proportional to the 3D property *z*, the length of the combined vector $\left({\hat{z}}_{t},\;{\hat{z}}_{s}\right)$ is also proportional to *z*. However, because the combined vector length depends on the individual cue strengths, it will fluctuate, with both nuisance variables affecting texture and shading (surface properties and environmental illumination). Critically, the central claim of our theory is that the goal of the visual system is to maximize sensitivity to underlying 3D information while minimizing sensitivity to nuisance variables.

In Appendix 1, we show that the combined estimate, calculated as a vector norm of single-cue signals scaled by parameters representing the variability of independent nuisance variables, achieves this goal. For the general case of multiple image signals this leads to Equation 3:

(3) $$\begin{array}{ccc} {\hat{z}}_{c}\; & = & \;\sqrt{{\left({\hat{z}}_{1}\right)}^{2}+{\left({\hat{z}}_{2}\right)}^{2}+...{\left({\hat{z}}_{n}\right)}^{2}}\\ & = & \;\sqrt{{\left(k_{1}z_{1}\right)}^{2}+{\left(k_{2}z_{2}\right)}^{2}+...{\left(k_{n}z_{n}\right)}^{2}}\; \end{array}$$

There are several nuances to the IC theory that should be noted. First, the value $\hat{z}$ does not necessarily imply a single depth value or depth map, but more generally can refer to any 3D property, such as slant or curvature map. Second, the cue strengths, *k_i_*, are not free parameters; they can be empirically measured from slopes of perceptual functions relating physical depth to perceived depth. Although the visual system must learn mappings from cue signals to perceived depth, it does not explicitly represent cue strengths. “Cue strength” refers to the empirically derived slope of the perceptual function. Third, because the model is additive, it may be misunderstood as producing systematic overestimations as more cues are added to a stimulus. In fact, it is quite the contrary. We speculate that removing cues brings the model outside of its optimal operating conditions, which results in underestimation of depth from reduced—or single-cue stimuli. We refer to Equation 3, which predicts an increase in perceived depth with the addition of cues as the vector sum model. Finally, although we have not yet proposed a learning model for how these perceptual functions are learned for single cues, it should be noted that this model is more parsimonious than the MLE model (which also has no specified learning mechanism) in two respects. First, this model does not assume that the visual system learns to extract unbiased estimates from individual cues. Second, it does not assume that the visual system requires a learning of the reliability for each individual cue across viewing conditions.

In summary, the primary difference between the two depth-processing models is how they handle nuisance variables. MLE models assume that depending on the “quality” (i.e., reliability) of 3D information specifying a given stimulus, 3D estimates will fluctuate from one view of the stimulus to the next. This fluctuation can be due to randomization in the stimulus generation or trial-by-trial “errors in the measurement of the gradient” (Hillis et al., 2004). For example, multiple views of stimuli carrying the same regular texture pattern of Figure 1b will produce a much smaller variation of 3D estimates than repeated viewing of the plaster texture of Figure 1c. When multiple cues are combined, the MLE model dynamically weighs the output of single-cue modules according to their individual “quality” to reduce response variability. Moreover, the MLE model assumes invariance of perceptual estimates by fiat because it postulates unbiased estimates. The goal thus is to simply increase precision of the estimate through cue combination. In contrast, the IC theory predicts only negligible fluctuations in estimates due to neural noise. Instead, fluctuation of depth estimates, despite the same distal structure, are due to changes in nuisance variables, such as changes in lighting conditions, viewing distance, etc. This can be readily seen in Figures 1a to 1c, where the same 3D surface is rendered under different surface properties and illumination conditions. The IC theory postulates that functions relating image signals to distal depth properties are learned in order to minimize the influence of changes in viewing conditions. The vector sum combination rule further increases sensitivity to changes in depth while being less sensitive to changes in nuisance variables. Although the aim of the MLE model is to reduce trial-by-trial variability around an unbiased estimate, the aim of the vector sum model is to reduce the influence of nuisance variables on the bias within the estimate. In other words, according to the IC theory, achieving invariance across viewing conditions and surface properties is a goal of the visual system, rather than an assumption.

The main goal of this study is to test the efficacy of the vector sum model in predicting several documented properties of depth perception while reinterpreting the mechanisms for cue processing and combination. Experiment 1 examines the inaccuracy of single-cue estimates and the systematic biases that can be expected when cues are combined. We show that these biases can be predicted without free parameters through the vector sum model. Experiment 2 replicates previous findings that discrimination thresholds decrease for combined-cue stimuli relative to single-cue stimuli. We discuss why the vector sum model and the MLE model make similar predictions regarding discrimination thresholds, but for very different reasons (i.e., reasons related to the properties of linear cue strengths vs. the properties of probability distributions). We then show that these thresholds can be predicted by the cue strengths measured in Experiment 1. Experiment 3 shows that increasing the reliability of flatness cues does not reduce perceived depth, following the vector sum model predictions. Furthermore, the results suggest that flatness cues are unlikely to allow the MLE model to account for the biases found in Experiment 1.

## Experiment 1

A first test of the IC theory is to verify that the combination of multiple cues leads to depth estimates in alignment with the vector sum model. According to Equation 3, the perceived depth of a combined-cue stimulus is predicted to be larger than the perceived depth of component cue stimuli. For the specific case of texture and binocular disparities Equation 3 can be reduced to the following equation:

(4) $$\begin{array}{c} {\hat{z}}_{c}=\sqrt{{\left(k_{t}z_{t}\right)}^{2}+{\left(k_{d}z_{d}\right)}^{2}}\; \end{array}$$

In single-cue conditions, only one cue varies in depth, which means that the cue strength of all other cues is zero. We studied the perceived depth of a sinusoidally corrugated surfaces by manipulating the amplitude of the sinusoids. In the disparity condition, the surface was specified by a random-dot stereogram (RDS) which did not provide any discernible texture information (i.e., *k_t_* = 0). In the texture condition, a compelling texture gradient specified the depth profile of the surface while binocular disparities were set to zero (*z_d_* = 0). In the combined-cue condition both texture and disparity information were present in the stimulus. The choice of having the texture stimulus be viewed binocularly was made for the practical reason of keeping the vergence signal constant in all viewing conditions, as monocular viewing may create the undesirable effect of perturbing egocentric distance information. This choice would be considered problematic from the perspective of the MLE approach because a null disparity field is a powerful cue to flatness. However, according to the vector sum model the absence of binocular disparities (*k_d_* = 0) or a null disparity field (*z_d_* = 0) should have the same effect on the perceived depth magnitude of a stimulus. This prediction, which is incompatible with the prediction of the MLE model, has been confirmed in a previous study that showed switching from monocular to binocular viewing of a 3D cylinder carrying texture information did not produce any change in perceived depth magnitude of the stimulus (Vishwanath and Hibbard, 2013). In Experiment 3 we further tested this hypothesis with the stimuli used in this experiment and confirmed this finding and the predictions of the vector sum model.

### Experiment 1: Methods

#### Participants

Eleven participants (three being the authors) were drawn from the Brown University community, and participants completed Experiment 1. Participants received either $12/hour or course credit as compensation. Participants provided informed consent prior to testing. The procedure reported was approved by the Brown University Institutional Review Board.

#### Apparatus

Experiments were completed on a Dell Precision T7500 (Dell, Inc., Round Rock, TX) powered by a NVIDIA Quadro 4000 graphics card (NVIDIA Corp., Santa Clara, CA). Stimuli were simulated on a Triniton GDM-f520 cathode-ray tube (CRT) monitor (Sony, Tokyo, Japan) with a resolution of 1024 × 786 at a refresh rate of 85 Hz. The display was projected onto a half-silvered mirror that was slanted 45° about the vertical axis in front of the participant with respect to the frontoparallel plane. The monitor was repositioned to different viewing distances using a linear actuator (Velmex, Inc., Bloomfield, NY). Binocular disparity was provided using NVIDIA 3D Vision 2 wireless glasses, which were synchronized to the refresh rate of the monitor to provide unique images to each eye. The interocular distance of every participant was measured using a digital pupillometer (Reichert, Inc., Depew, NY). Participants viewed the stimuli while positioned on a chinrest.

#### Stimuli

The target stimuli were 3D corrugated surfaces whose depth profile followed a sinusoidal modulation along the vertical axis. The corrugated surfaces were seen through a virtual square frame such that the surface subtended approximately 8° of visual angle. This frame was used to eliminate contour information indicating a depth modulation. The wave period was 4.50° of visual angle.

Participants made depth judgments by adjusting a 2D sinusoidal probe whose horizontal amplitude varied along the vertical axis. The wave period of the probe also subtended 4.50° of visual angle. The phase of the 3D surface was randomly varied on each trial to eliminate depth adaptation. However, the phase of the probe line remained constant throughout all sessions.

**Figure 2.**
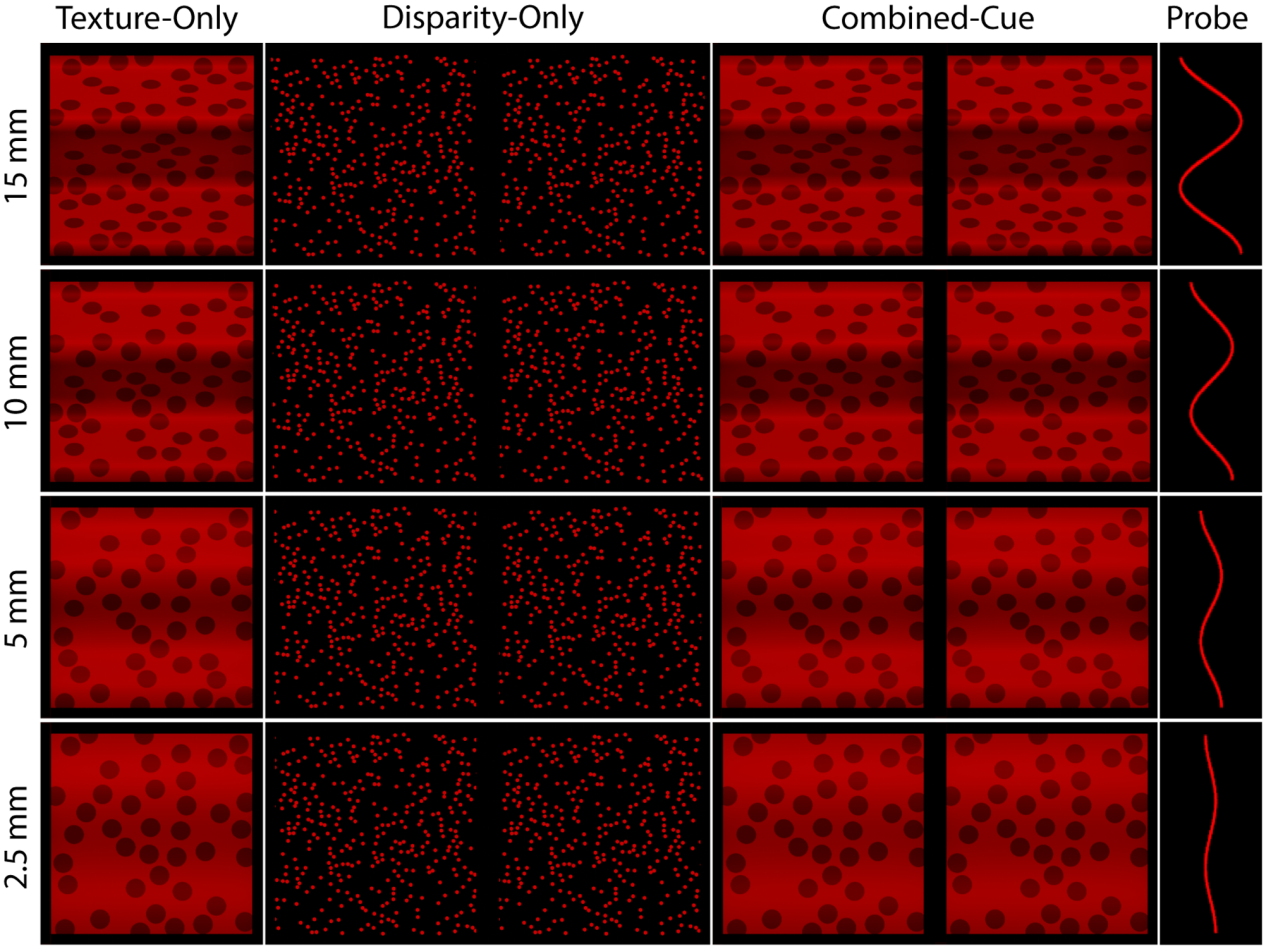
Examples of stimuli defined by each cue type at each depth for a fixation distance of 40 cm. In the texture-only condition, the 3D structure was defined by polka dots with shading added to eliminate depth order ambiguities. Disparity-only defined surfaces were random-dot stereograms (cross-fusable). In the combined-cue condition both cues were present (cross-fusable). Participants adjusted the amplitude of a 2D probe to report perceived depth.

Participants judged the depth of surfaces from three cue conditions: texture-only, disparity-only, and combined-cue. Figure 2 shows the cue conditions (columns) for each simulated depth magnitude (rows) at a fixation distance of 40 cm. The last column shows the probe set at the correct amplitude. Texture-only defined surfaces were constructed by volumetric texturing. Centers of spheres with radii subtending a visual angle of 0.55° were placed onto the simulated 3D corrugated surface. Any vertex of the wave that intersected a sphere was painted black. This produced a compelling texture gradient on the image projection. To eliminate depth order ambiguity, shading information was produced by placing a single directional light source angled 45° around the *x*-axis above the surface. Although we added a shading component, we refer to this cue condition as the texture condition for simplicity and to avoid confusion with the experimentally defined combined-cue condition. Because the two cues are consistent with each other, both the MLE model and vector sum model consider the texture-shading stimulus as a single-cue stimulus. Furthermore, we chose to use a polka dot texture because it has been shown to elicit the highest levels of precision in discrimination tasks when compared with other texture patterns (Rosas, Wichmann, & Wagemans, 2004). This means that it will have the largest potential to influence the depth percept due to being the most reliable (MLE model) or having the largest cue strength (vector sum model) relative to other textures. Additionally, polka dots have been used extensively by other researchers in the study of 3D from texture (Chen & Saunders, 2020; Knill, 1998a; Knill, 1998b; Todd & Thaler, 2010). To keep a steady fixation at the center of the display as in the disparity-only and combined-cue conditions, texture-only stimuli were also seen binocularly.

Disparity-only defined surfaces (texture and shading specified zero depth) were presented as a RDS consisting of 400 dots each kept at a constant visual angle of 0.1° to remove compression and scaling information. The dots were uniformly distributed on the image plane with the constraint that they did not overlap. Because part of the surface was occluded by a virtual frame, there were on average 320 visible dots. Two views were rendered by placing the rendering cameras at the estimated locations of the observer's nodal points, which were determined after measuring for each observer the interocular distance. NVIDIA 3D Vision 2 wireless stereo-glasses were used to separate the projection of the left and right images for the appropriate eye. Combined-cue stimuli were obtained by stereoscopically rendering the polka dot textured surfaces.

#### Procedure

Participants completed two blocks within a single session. Each block had a constant fixation distance of either 40 or 80 cm. Within each block, participants viewed sinusoidal surfaces with four different peak-to-trough depths (2.5, 5, 10, or 15 mm) defined by one of three cue types (disparity-only, texture-only, and combined-cue), with seven repetitions for each combination of depth and cue type. Thus, each block recorded 84 judgments and lasted approximately 20 minutes. Three participants (all authors) completed only five repetitions for each condition. Excluding these participants does not change the pattern of results. At the onset of each trial, a fixation cross was displayed for 700 ms, followed by the presentation of the surface stimulus, as well as a 2D sine wave probe at the bottom of the display. Participants adjusted the amplitude of the probe until the peak-to-trough length matched their perceived depth of the target stimulus. During the adjustment they were free to move their eyes back and forth between the 3D surface and the 2D probe. Once they were satisfied with their setting, they submitted their judgment with a button press, which also initiated the next trial. Before the experimental session, participants completed a small number of practice trials with stimuli of random depths. No feedback about response accuracy was provided at any point.

### Experiment 1: Results and discussion

**Figure 3.**
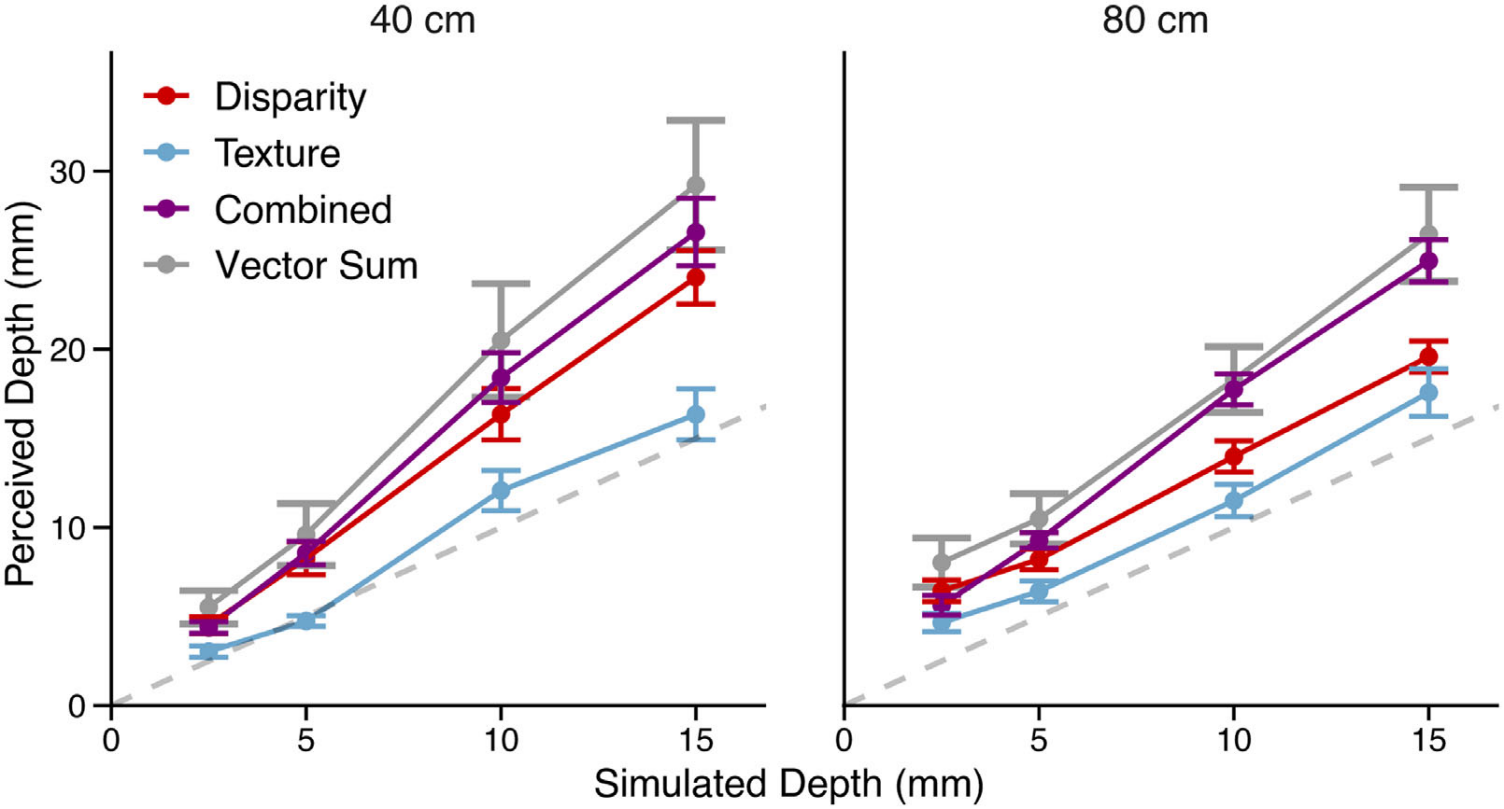
Average depth judgments as a function of simulated depth for viewing distances of 40 cm (left panel) and 80 cm (right panel) with error bars for the standard error of the mean. The horizontal axis labeled “Simulated Depth” represents the peak-to-trough depth of the corrugated 3D surface, and the vertical axis labeled “Perceived Depth” represents the set amplitude of the 2D probe. The different cue conditions are denoted by the color of the data points: purple for the combined cue condition, red for the disparity condition, and blue for the texture condition. The vector sum prediction is denoted by the gray line with 95% confidence intervals at each point. The dashed gray line is the unity line denoting veridical perception.

Qualitatively, the vector sum model predicts that the combined-cue stimulus should be perceived deeper than the single cue stimuli. Figure 3 shows the average probe settings across cues (denoted by line color) and fixation distances (denoted by separate panels). A repeated-measures analysis of variance (ANOVA) found a main effect of simulated depth, *F*(1, 10) = 272.67, *p =* 1.4e-8, generalized η^2^ = 0.89, and cue type, *F*(2, 20) = 48.40, *p* = 2.2e-8, generalized η^2^ = 0.43. For both fixation distances, the perceived depth of combined-cue stimuli (purple diamonds) was consistently greater than the perceived depth of single-cue stimuli (red squares, blue circles). A Bonferroni-corrected post hoc analysis confirmed that perceived depth in the combined-cue condition was larger than the perceived depth in both the disparity condition, *t*(10) = 4.42, *p* = 0.0039, and the texture condition, *t*(10) = 10.59, *p* = 2.8e-6. Additionally, texture stimuli were in general perceived as shallower than disparity stimuli, demonstrating cue-specific biases, *t*(10) = –5.02, *p* = 0.0016.

All interactions were significant. The interactions between cue type and fixation distance, *F*(2, 20) = 3.76, *p* = 0.041, generalized η^2^ = 0.037, between simulated depth and fixation distance, *F*(1, 10) = 7.19, *p* = 0.023, generalized η^2^ = 0.07, and between all three factors, *F*(2, 20) = 5.11, *p* = 0.016, generalized η^2^ = 0.031, reflect the dependence of cue strength on how the fixation distance influences the quality of the cue. This was expected particularly for the disparity cue where a lack of depth constancy across distances is a well-documented phenomenon (Johnston, 1991). The interaction between simulated depth and cue type, *F*(2, 20) = 45.42, *p* = 3.7e-8, generalized η^2^ = 0.20, further supports the existence of cue-specific biases due to differing cue strengths between cue types.

The vector sum model predicts that the perceived depth of the combined cue stimulus should be the square root of the sum of squares of the perceived depth of the single-cue stimuli (Equations 3 and 4). Figure 3 plots the average predictions of the vector sum model across participants with 95% confidence intervals (gray). Given that we assume, under ideal conditions, a linear mapping, the model can directly predict the cue strength of the combined-cue from those of the single cues through Equation 4. The prediction is simplified to the following because the simulated depth rendered for each cue is the same (i.e., there are no cue conflicts):

(5) $$\begin{array}{c} {\hat{z}}_{c}=\sqrt{{\left(k_{t}z\right)}^{2}+{\left(k_{d}z\right)}^{2}}=\sqrt{{k_{t}}^{2}+{k_{d}}^{2}}\;z=\;k_{c}z\; \end{array}$$

**Figure 4.**
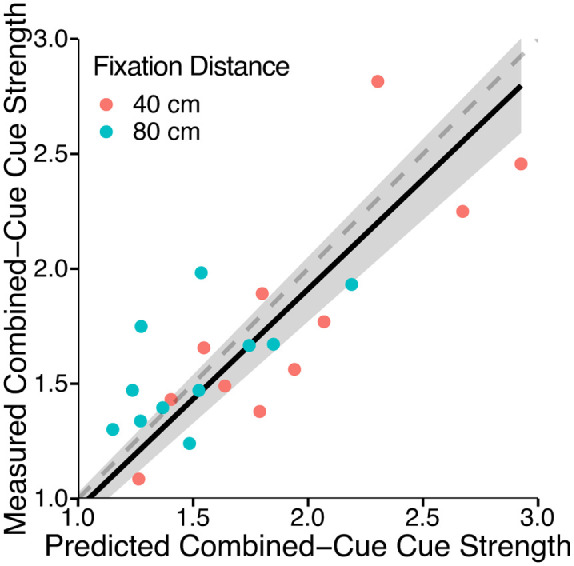
Observed combined cue strength versus predicted combined cue strength. The predicted combined cue strength is computed with the vector sum model without free parameters directly from the single-cue strengths. The single-cue and combined-cue strengths were determined by the slopes of linear fits. Each data point represents a subject either in the 40 cm (red) or 80 cm (blue) fixation distance condition. The dashed gray line represents accurate predictions. The gray area denotes the 95% confidence interval of the linear fit (black line) of the observed versus the predicted cue strength.

Because the empirically measured slopes of the functions mapping distal depth to the reported perceived depth are proxies for the cue strengths, Equation 5 predicts the slope of the combined-cue estimate, *k_c_*, from the slopes of the single-cue estimates ($k_{c}=\sqrt{{k_{t}}^{2}+{k_{d}}^{2}}$). Figure 4 shows the predicted slopes plotted against the measured slopes for each participant. Without fitting any free parameters, the linear fit of measured versus predicted slopes with the intercept set to 0 closely matches the unity line (slope = 0.96 with *SE =* 0.034). Although the correlation coefficient of this linear fit is not very high (*r* = 0.79), the predictive power of the vector sum model is superior to that of the MLE model, which would require the additional assumption of flatness cues to explain the data. Without assuming the role of unmodeled flatness cues, the results clearly contradict the MLE model prediction that the perceived depth of the combined-cue stimulus falls in between the perceived depth of the single-cue stimuli. Because the reliability of the flatness cues in these particular displays is unknown, a fit of the MLE model would therefore require free parameters modeling the variance of the noise of the flatness cues. In this case, the MLE model would be less parsimonious than the vector sum model. Furthermore, although the MLE model predictions may be amended by introducing flatness cues, we will provide evidence in Experiment 3 rejecting the flatness cues explanation. Additionally, single-cue and combined-cue depths were consistently overestimated in five of six stimulus conditions, contradicting the veridicality assumption of the MLE model.

If previous findings from ostensibly similar tasks have supported the MLE model (Hillis et al., 2004; Knill & Saunders, 2003; Lovell, Bloj, & Harris, 2012), then why does the MLE model fail in predicting these results? A critical difference is that observers in this task provided absolute judgments of depth using a probe figure, whereas in earlier studies observers made relative judgments by comparing or matching two 3D shapes. Although relative judgment tasks are often useful, they cannot reveal systematic biases in depth perception. For example, Hillis et al. (2004) asked participants to match the perceived slant of an adjustable cue-consistent surface with the slant of a fixed cue-conflict surface (i.e., the simulated slants from texture and from disparity were either matched or mismatched). The cue-consistent slant that yielded a match was predicted through the MLE model (Equation 1). However, there is no guarantee that either surface was perceived veridically. Nevertheless, it is notable that discrimination thresholds measured on single-cue stimuli were indeed good predictors of the weights estimated in the slant matching task. The IC theory, however, provides a radically different interpretation of discrimination thresholds. When this new interpretation is adopted, it can be shown that an approximation of the vector sum model makes identical predictions of the results of Hillis et al. (2004) to those of the MLE model (Appendix 2).

#### Cue uncertainty and judgment variance

**Figure 5.**
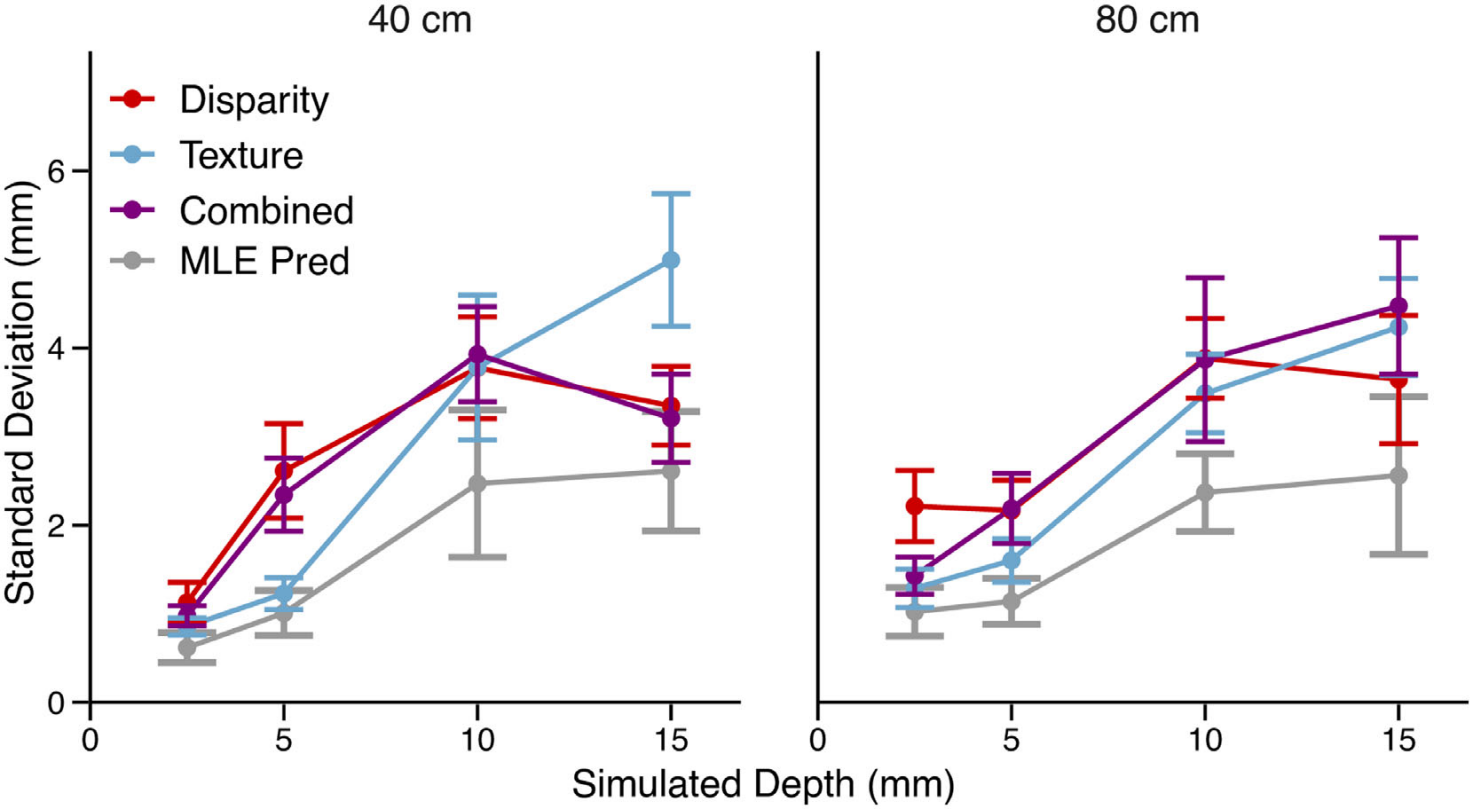
The average standard deviations of the probe adjustment task in Experiment 1 with error bars averaged across subjects. The MLE predictions together with the 95% confidence intervals are shown in gray. The patterns of the *SD*s do not align with the MLE predictions because the *SD*s observed in the combined-cue condition are not smaller than the *SD*s observed in the single-cue condition.

An important prediction of the MLE model is that the variance of the combined-cue estimate should be smaller than the variances of the single-cue estimates (Equation 2). Tests of this MLE prediction are usually conducted by measuring discrimination thresholds of single-cue and combined-cue stimuli. However, noise coming from depth estimation should also surface in the standard deviation of probe adjustments. We should therefore expect that the standard deviation of probe adjustments in the combined-cue condition should be smaller than that measured in the single-cue conditions. Alternatively, the vector sum model assumes that depth estimates are approximately deterministic, only affected by negligible neural noise. According to this theory, variability in perceptual judgments is all due to late-stage, task-related processes independent of the stimulus itself. In this task, participants were required to represent the amount of depth they perceived through the width of the probe. The IC theory predicts that the magnitude of the noise observed in the observer's settings is only dependent on this specific task and it is not influenced by the cues present in the display. We therefore expect that there is no difference between the cue types in the *SD* of the probe adjustments. Given the different predictions of the two models, we tested whether there was a difference in the *SD* between the cues. Figure 5 shows the *SD* of the probe adjustment task as function of simulated depth in all experimental conditions. In this figure the prediction of the MLE model for the *SD* of the combined-cue adjustments is shown in gray.

A repeated-measures ANOVA indicated one main effect of simulated depth, *F*(1, 10) = 54.75, *p* = 2.3e-5, generalized η^2^ = 0.57. This follows the classic effect of Weber's law where the response variance is proportional to the magnitude of the stimulus, in this case the surface depth. There was also an interaction between the cue type and simulated depth, *F*(2, 20) = 7.31, *p* = 0.0041, generalized η^2^ = 0.090. However, there was no main effect of cue type, *F*(2, 20) = 0.45, *p* = 0.65, generalized η^2^ = 0.0053. This can be observed in Figure 5 where the combined-cue standard deviation (purple) is not smaller than the single-cue standard deviations, as predicted by the MLE model (gray). Instead, these results support the prediction of the vector sum model that noise observed in perceptual judgments is stimulus independent. Because the vector sum predicts a null effect of cue type, we conducted a Bayes factor analysis using the BayesFactor package in R (R Foundation for Statistical Computing, Vienna, Austria) (Morey & Rouder, 2021). A Bayes factor of 0.055 indicated strong evidence for a model including fixed effects of simulated depth and fixation distance compared with a model including the same fixed effects with the inclusion of cue type. Both models included a random effect for participants.

These results are particularly intriguing because they seem to be inconsistent with findings obtained in experiments where discrimination thresholds are used to test the predictions of the MLE model. As we will show, discrimination thresholds measured from the same stimuli show that the thresholds for the combined-cue stimuli are smaller than those of the component cue stimuli as predicted by Equation 2. We will also show that the vector sum model makes the same predictions but through an entirely different mechanism.

## Experiment 2

**Figure 6.**
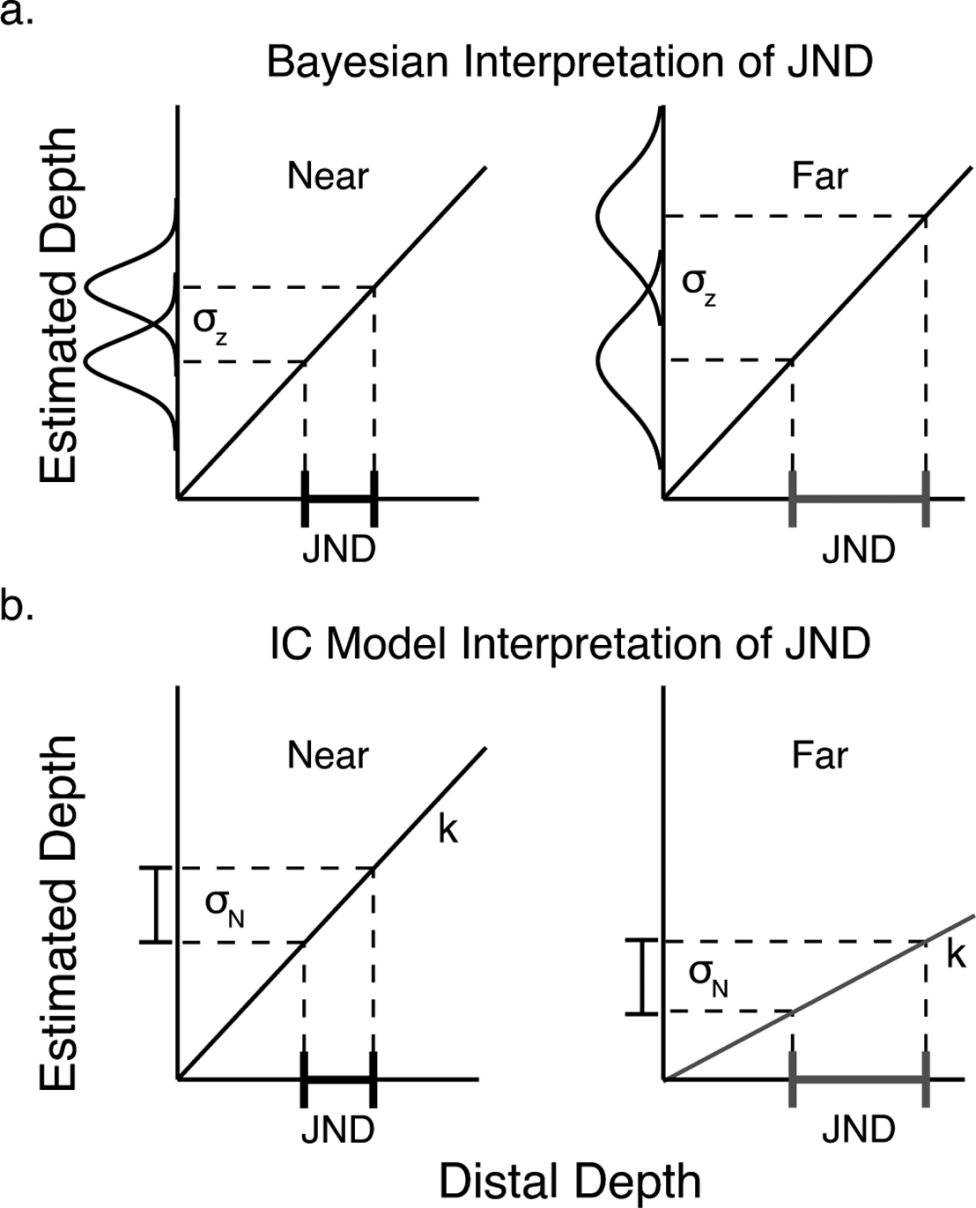
Two different interpretations of the JND according to the MLE model (a) and the IC theory (b). (a) MLE models assume that variability of depth judgments is due to uncertainty of 3D estimates. For example, disparities at near distances (left) are more reliable than disparities at far distances (right). Therefore, the distribution of depth estimates is narrower at near distances than at far distances. In the example, only a small change in distal depth is necessary to overcome the perceptual noise at near distances. However, at far distances a larger change is needed. Note that the function relating distal depth to estimated depth is veridical. (b) The IC theory assumes approximately deterministic estimates. It also predicts that the main cause of variability of perceptual judgments is task related. For the IC theory, the JND measures the depth difference needed to overcome the task-related noise. When the cue strength is large, as what happens for disparity fields at near distances, only a small distal depth difference is needed. When the cue strength is small, as it is for disparity fields at far distances, a larger distal depth difference is needed.

The central hypothesis of the MLE framework is that cue combination leads to an increase in the reliability of the depth estimate. In many previous investigations, the reliability of a depth estimate has been assumed to be directly reflected by the just noticeable difference (JND) in a two-interval forced-choice (2IFC) task. The JND is a discrimination threshold measure taken as the difference in distal depth that leads to 84% accuracy in identifying the deeper stimulus. Under the MLE model, this is interpreted as the standard deviation of the noise in the estimation process. Figure 6a depicts how typical MLE models assume that JNDs arise from a noise-free decision process that compares two noisy estimates. For example, the JNDs are larger for a disparity stimulus at near viewing distances than at far viewing distances due to less estimation noise. Studies using this approach have repeatedly demonstrated that single-cue and combined-cue JNDs adhere to the relationship predicted by the MLE model (Equation 2) (Ernst & Banks, 2002; Hillis et al., 2004; Knill & Saunders, 2003).

In contrast, the IC theory assumes that the noise in the estimation process is negligible. In other words, perceived depth is approximately the same across repeated viewings of the same stimulus under the same viewing conditions. However, noise in the response distributions of a task (task noise, which is often considered negligible by MLE models of cue combination) may arise due to factors such as response execution and memory requirements. Importantly, this noise is independent of distal stimulus properties such as texture quality or viewing distance. This leads to a different interpretation of the JND: Given a particular cue strength, the JND is the change in distal stimulus magnitude needed to produce a perceptual difference that is large enough to overcome the effects of task noise which we represent as σ*_N_*. As shown in the hypothetical experiment of Figure 6b, the JND is larger at the far viewing distance because the cue strength becomes weaker (consistent with the fact that binocular disparities and their gradients decrease with viewing distance). We see that the JND is inversely proportional to the cue strength ($J_{i}\;=\;\frac{\sigma_{N}}{k_{i}}$). Recall that the vector sum model posits that adding cues to a stimulus increases the combined-cue strength according to the magnitude of the vector of cue signals. Because the JND is inversely proportional to cue strength, the vector sum model therefore predicts that the JND shrinks with additional cues, similar to the MLE model. Specifically, the texture-only, disparity-only, and combined-cue JNDs are given by $J_{t}=\frac{\sigma_{N}}{k_{t}}$ , $J_{d}=\frac{\sigma_{N}}{k_{d}}$, and $J_{c}=\frac{\sigma_{N}}{k_{c}}=\frac{\sigma_{N}}{\sqrt{{k_{t}}^{2}+{k_{d}}^{2}}}$, respectively. Appendix 3 shows how, from these equations, we can predict the combined-cue JND directly from the single-cue JNDs as follows: $J_{c}=\frac{1}{\sqrt{\frac{1}{J_{t}^{2}}+\frac{1}{J_{d}^{2}}}}$. Notice that this equation is formally identical to Equation 2 of the MLE model, where JNDs are assumed to measure the estimation noise (i.e., *J_i_* = σ*_i_*). However, the vector sum model predicts that this relationship will hold at the same *perceived depth,* where task-related task noise is expected to be equivalent as the decision process operates on perceived depth, whereas the MLE model predicts it will hold at the same *simulated depth*, where estimation noise is expected to be equivalent. Thus, the prediction of the two models for a given dataset may slightly differ, as we will show.

The goal of Experiment 2 was to demonstrate that the vector sum model correctly predicts the relationship between single-cue and combined-cue JNDs for the same stimuli presented in Experiment 1. Additionally, we aimed to show the close relationship between the magnitude of JNDs and the independently measured cue strengths obtained in Experiment 1. These findings demonstrate that the interpretation of the JND by the IC theory is highly consistent with empirical results of depth discrimination tasks.

### Experiment 2: Methods

#### Participants

Eight participants from Experiment 1 returned to complete Experiment 2, including two of the authors.

#### Stimuli

**Figure 7.**
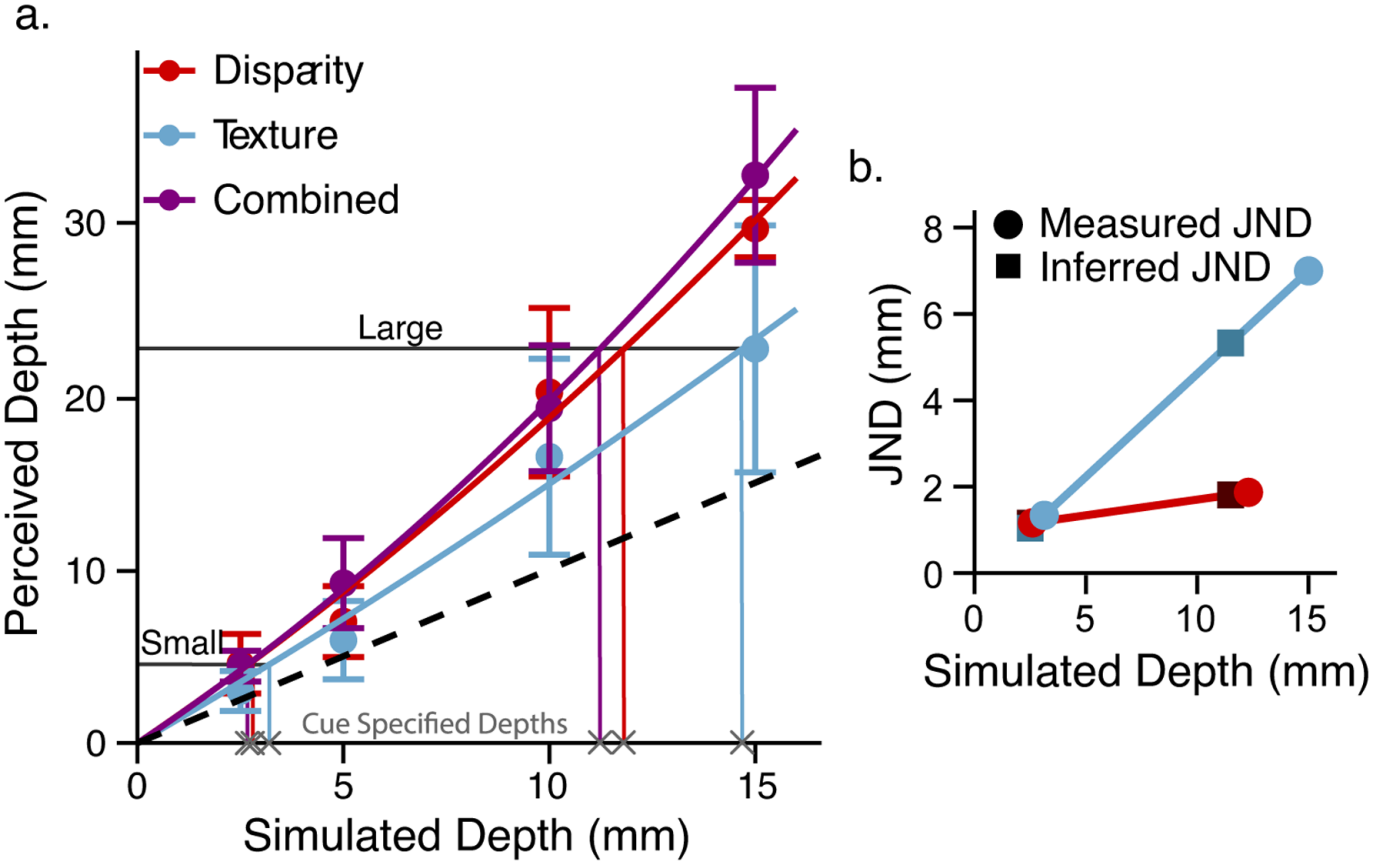
(a) An example from a representative observer of how simulated depths for the fixed standards were chosen at 40-cm fixation distance. Two perceived depths were chosen to generate large and small pairs of standard cue-specified depths. For a given perceived depth, each cue requires a different simulated depth to elicit that same perceived depth. These unique simulated depths were inferred for each cue through the intersection between curvilinear fits to the data (solid curved lines) and horizontal lines at the set perceived depths. The vertical lines indicate these inferred values. (b) MLE predictions require JNDs measured at the same simulated pedestal depth values. However, we measured the JNDs at slightly different pedestal values (circles). We therefore inferred the JNDs at the required standard values through interpolation or extrapolation (squares).

The stimuli were identical to those in Experiment 1. However, in this experiment participants did not provide a judgment of absolute perceived depth. Instead, they performed a 2IFC depth discrimination task. To make quantitative predictions of JNDs from the vector sum model, the perceived depth must be matched across the single-cue and combined-cue standards so that the task noise, which is dependent on perceived depth, is kept constant. Thus, we used data from Experiment 1 to infer, for each participant in each viewing condition, a set of three simulated depths for texture-only, disparity-only, and combined-cue stimuli that elicited two perceived depths (Figure 7a, horizontal lines). These simulated depths served as the standard stimuli in the 2IFC tasks, around which the JND was measured. For each viewing distance, we defined a large standard and small standard. The perceived depth that defined the small standard was anchored by the cue that elicited the greatest response at a distal depth of 2.5 mm. For the representative participant depicted in Figure 6a, the small standard corresponded to a perceived depth of approximately 4.5 mm, as this was the greatest reported perceived depth at 2.5 mm of simulated depth. Similarly, the simulated depth values for the large standard stimuli were anchored by the smallest perceived depth for a simulated depth of 15 mm. The simulated depth values for the various standard stimuli were chosen by interpolation using second-order curvilinear fits (see Figure 7a). Through this procedure we determined 12 standard stimuli (3 cues × 2 viewing distances × 2 perceived depths) to be used in a 2IFC depth discrimination task.

#### Procedure

Participants performed a 2IFC task in which the perceived depth of a standard stimulus with a fixed simulated depth was compared with that of a comparison stimulus whose simulated depth was varied through a staircase procedure. Four staircases were used in each condition (2-up/1-down, 1-up/2-down, 3-up/1-down, and 1-up/3-down) with 12 reversals each. On each trial, a fixation cross was displayed (700 ms), followed by the first stimulus (1000 ms), followed by a blank screen (1000 ms), then, again, the fixation cross (700 ms), and finally the second stimulus (1000 ms). Participants then reported with no time constraint which surface was perceived as having greater peak-to-trough depth through a keypress.

Response data were analyzed using a psychometric analysis package (Wichmann & Hill, 2001) in MATLAB (MathWorks, Natick, MA). The data from each staircase procedure were fit with a cumulative Gaussian function. The point of subjective equality (PSE) was defined as the simulated depth at which participants responded with 50% accuracy. The JND was defined as the difference between the PSE and the simulated depth at which participants responded with 84% accuracy.

#### Experiment 2: Results and discussion

**Figure 8.**
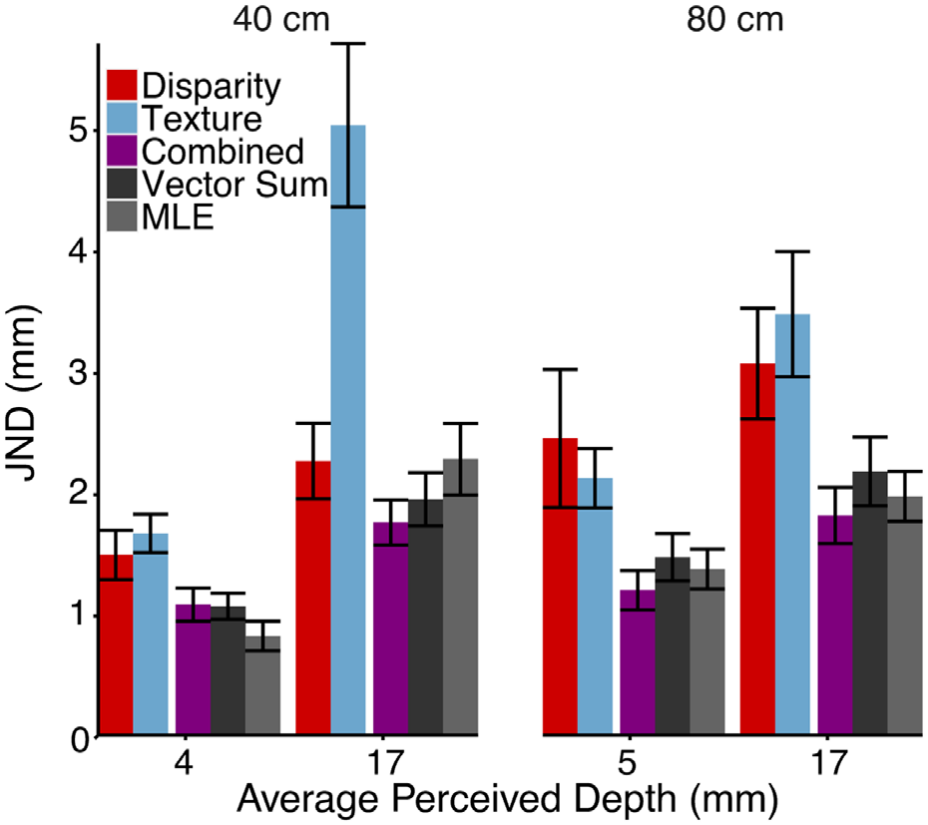
The JND averaged across participants along with model predictions. The horizontal axis displays the average perceived depth of the standard. The perceived depth of the standard was chosen uniquely for each subject based on procedures stated in the previous section (see Figure 7a). The vertical axis represents the JND in millimeters. Red, blue, and purple indicate JNDs measured for the disparity, texture, and the combined cue, respectively. Dark gray represents the vector sum model predictions, and light gray represents MLE predictions. Error bars show the standard error around the between-subject averages.

Figure 8 (colored bars) shows the average JND in each stimulus condition. On the horizontal axis, we indicate the average perceived depth corresponding to the two standard stimuli at each viewing distance. A repeated-measures ANOVA reported a significant main effect of cue, *F*(2, 14) = 25.42, *p* = 2.2e-5, generalized *η*^2^ = 0.41. A critical prediction of both the MLE and vector sum model is that the combined-cue condition elicits a smaller JND than the single-cue condition. Bonferroni-corrected *t*-tests confirmed that the JND for the combined-cue stimuli (purple) was smaller than the JND for the disparity (red), *t*(7) = –4.60, *p* = 0.005, and texture stimuli (blue), *t*(7) = –7.93, *p* = 1.9e-4, conditions. Additionally, we found a significant main effect of perceived depth, *F*(1, 7) = 55.54, *p* = 1.4e-4, generalized η^2^ = 0.38, with JNDs increasing for larger perceived depths. We suspect that this may be due to a form of Weber's law where the noise from the encoding and decoding of perceived depth to and from memory depends on the magnitude of perceived depth. We explore the implications of Weber's law further in the next section.

We also found significant interactions between perceived depth and viewing distance, *F*(1, 7) = 8.17, *p* = 0.024, generalized η^2^ = 0.052, between cue type and perceived depth, *F*(2, 14) = 11.31, *p* = 0.0012, generalized η^2^ = 0.20, and across all three factors of cue type, perceived depth, and viewing distance, *F*(2, 14) = 5.54, *p* = 0.017, generalized η^2^ = 0.074. These interactions, similar to Experiment 1, suggest a dependence of the cue strength on the cues and their viewing conditions. However, the key result is that the combined-cue JND is smaller than the single-cue JND in all conditions. Although this is often taken as evidence for the MLE model, here we show that it can also be predicted by the vector sum model.

The gray bars in Figure 8 show the predictions of the vector sum model (dark gray) and the MLE model (light gray) for the combined-cue JND. Recall that the vector sum model predictions are based on the single-cue JNDs for standard simulated depths that elicit the same *perceived depth* as the combined-cue stimulus. Equating the perceived depths of the standards was expected to approximately match the task noise across the three cue conditions. In contrast, the MLE model predictions are based on the single-cue JNDs for single-cue stimuli with the same *simulated depths* as the combined-cue stimulus. Although we did not measure the single-cue JNDs at fixed simulated depths, Figure 7b demonstrates how, for each participant, we inferred the appropriate values for the MLE model (squares) from linear fits of the measured JNDs (circles). Regardless, in Figure 8 we see that the predictions for the two models are very similar, as should be expected, with no significant difference in accuracy, *t*(7) = –0.39, *p* = 0.71.

#### Relationship between JND and cue strength

The IC theory contends that the JND is not a measure of estimation noise, which is assumed to be negligible, but rather the noise that emerges from task-related demands and the cue strength of the varying comparison stimulus. In a 2IFC task, the task demands lie in the remembering of two perceived depths across a time interval (e.g., temporal decay in memory). The cue strength of the comparison is integral because it determines how much change in the simulated depth of the comparison is necessary to produce a perceived depth difference large enough to overcome the task noise, σ*_N_* (Figure 6b). Furthermore, we expect that the JND is susceptible to a form of Weber's law on perceived depth, where increases in perceived depth will cause an increase in the standard deviation of the task noise. If we therefore assume that σ*_N_* increases with the perceived depth, ${\hat{z}}_{s}$, of the standard stimulus through a Weber fraction, *W*, then $\sigma_{N}=W{\hat{z}}_{s}+c$, where *c* is a constant reflecting a baseline noise. Because $J_{ij}\;=\frac{\sigma_{N}}{k_{ij}}$, where *k_ij_* is the cue strength of cue *i* (disparity-only, texture-only, and the combined-cue) for viewing condition *j* (40 cm and 80 cm fixation distance), we can obtain $J_{ij}=\frac{W{\hat{z}}_{s}+c}{k_{ij}}$. Because the perceived depth of the standard is ${\hat{z}}_{s}=k_{ij}z_{s}$, where *z_S_* is the distal depth of the standard stimulus, the JND can be modeled relative to the distal depth by Equation 6:

(6) $$\begin{array}{c} J_{ij}=Wz_{s}+\frac{c}{k_{ij}}\; \end{array}$$

**Figure 9.**
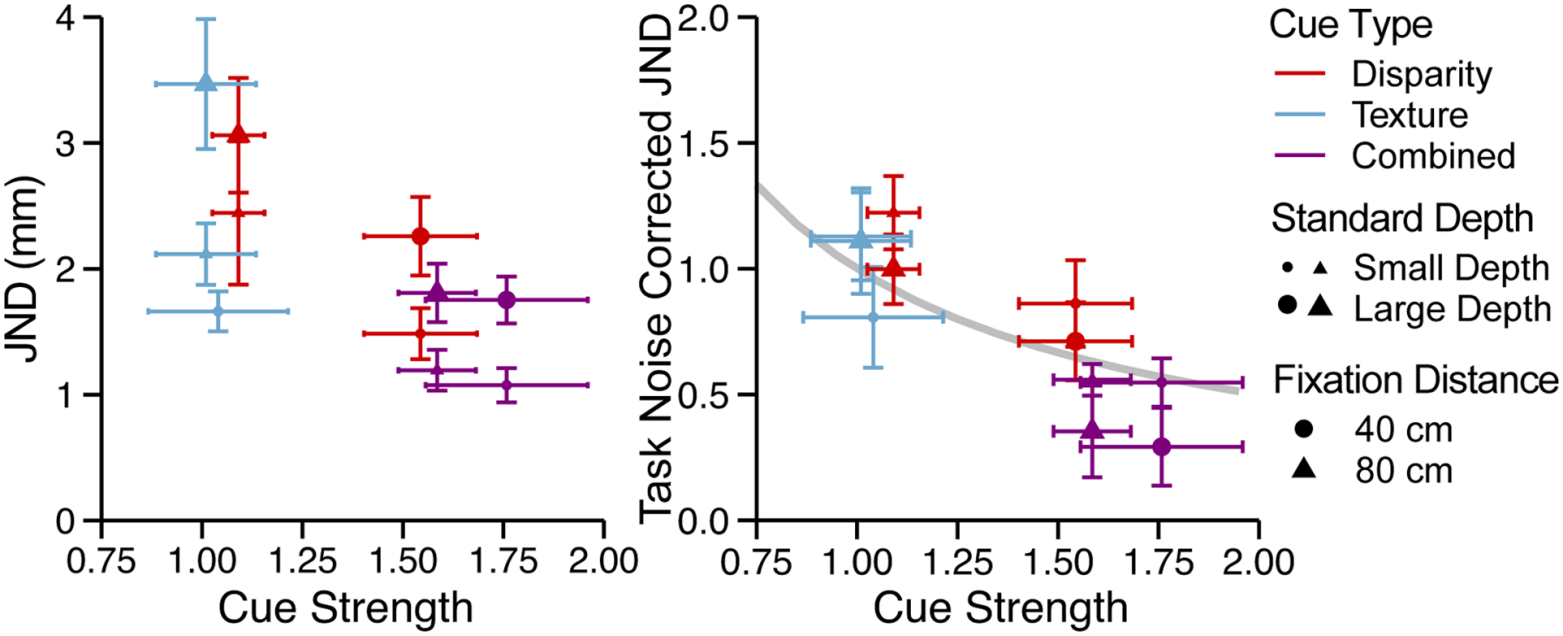
(a) The JND against the cue strength extracted from Experiment 1 averaged across participants with error bars representing the *SE*. There is an inverse relationship between cue strength and the JND. (b) Task noise-corrected JNDs plotted against the cue strength. The vector sum model predicts that there should be a hyperbolic relationship between the JND and the cue strength, which is plotted by the gray curve.

We expect that the JND depends (1) on the magnitude of the perceived depth of the standard, and, most critically, (2) on the cue strength, *k_ij_*, of the comparison. We set, for each participant, cue strength *k_ij_* to the individual slopes from linear fits mapping the simulated depths observed in Experiment 1 to the perceived depths. To infer the Weber fraction and the noise coefficient, we fit Equation 6 to the estimated JNDs of each participant. We found both the Weber fraction (*M* = 0.13 mm, *SE* = 0.031 mm) and the noise coefficient (*M* = 1.66 mm, *SE* = 0.36 mm) were significantly greater than 0, *t*(7) = 4.11, *p* = 0.0045 and *t*(7) = 4.57, *p* = 0.0026, respectively. Critical here is that the JND measured in Experiment 2 depends on the cue strength seen in Experiment 1 (Figure 9a). Using Equation 6 for each participant, we can discount from the observed JND the contribution of the Weber law and the noise constant to produce a noise corrected JND ($\frac{J_{ij}-Wz_{s}}{c}$). Figure 9b plots the relationship between the cue strength and corrected JND averaged across participants. Horizontal error bars indicate the variability of the cue strength across participants and vertical error bars the variability of the corrected JND across participants. When the Weber fraction and the noise constant are factored out, the JND is shown to be dependent on the cue strength (1/*k_ij_*) and independent of the cue type as predicted by the MLE model. For example, the JND of the disparity stimulus at the close viewing distance (Figure 9b, red circles) is smaller than the JND at the larger viewing distance (Figure 9b, red triangles) because the strength of disparity at the smaller viewing distances is larger than the strength of disparity at the larger viewing distance.

Finding a direct relationship between the cue strengths measured in the absolute judgment task of Experiment 1 and the JNDs also provides support for the 2IFC task as a tool to measuring depth perception. Studies in the past have provided evidence that discriminating between two surfaces may be completed through comparing image level variations, such as texture compression, rather than comparing their 3D percept (Todd et al., 2010). By showing that the thresholds can be predicted by data from an absolute judgment task, we confirm that participants were performing comparisons through their interpretation of the depth of the object rather than image-level features.

It should be noted that the condition for the large texture standard (average perceived depth of 17 mm) at 40-cm fixation distance was removed from this analysis. We noticed that the JND in this condition was much larger than in the other conditions and, therefore, it was possibly an outlier (Figure 8). We confirmed this by performing a type 10 Grubbs test on the average JNDs and found that it passed the significance test as an outlier (*p* = 0.019). We expect the poor JND is due to limitations set by the small wavelength at the near fixation distance. Deeper surfaces will cause the surface between the peak and trough to asymptotically approach a planar slant, which has been shown to provide poor texture gradients at small visual angles (Knill, 1998b; Todd & Thaler, 2010).

In summary, these results indicate that the JND in a 2IFC task can be explained by the cue strength and not by the noise of depth estimates. This finding aligns with the predictions of the IC theory that postulates a deterministic mapping of depth modules between distal 3D properties and the module outputs.

## Experiment 3

The main aim of this experiment was twofold. The first aim was to test another difference in predictions between the MLE and vector sum model regarding the presence of cues that specify a frontoparallel surface, termed flatness cues. The second aim was to justify the use of binocular viewing of texture-only stimuli in Experiment 1. By considering the role of flatness cues, the MLE model could accommodate the finding that combined-cue stimuli are perceived as deeper than single-cue stimuli. Proponents of the MLE theory argue that, when stimuli are rendered on flat displays, experimenters typically fail to eliminate all uncontrolled depth cues. As a result, residual depth information (e.g., blur gradient) may specify the flat surface of the screen (Watt, Akeley, Ernst, & Banks, 2005). If flatness cues influence depth judgments, then single-cue conditions are inadvertently testing the combination of the single-cue and flatness cues. In this case, the MLE model predicts that the combined-cue stimulus may be perceived as deeper than the single-cue stimuli. On the other hand, according to the vector sum model, flatness cues should have no influence on perceived depth because they specify zero depth and thus do not contribute to the vector sum. Thus, it predicts Experiment 1 without the need for fitting parameters.

**Figure 10.**
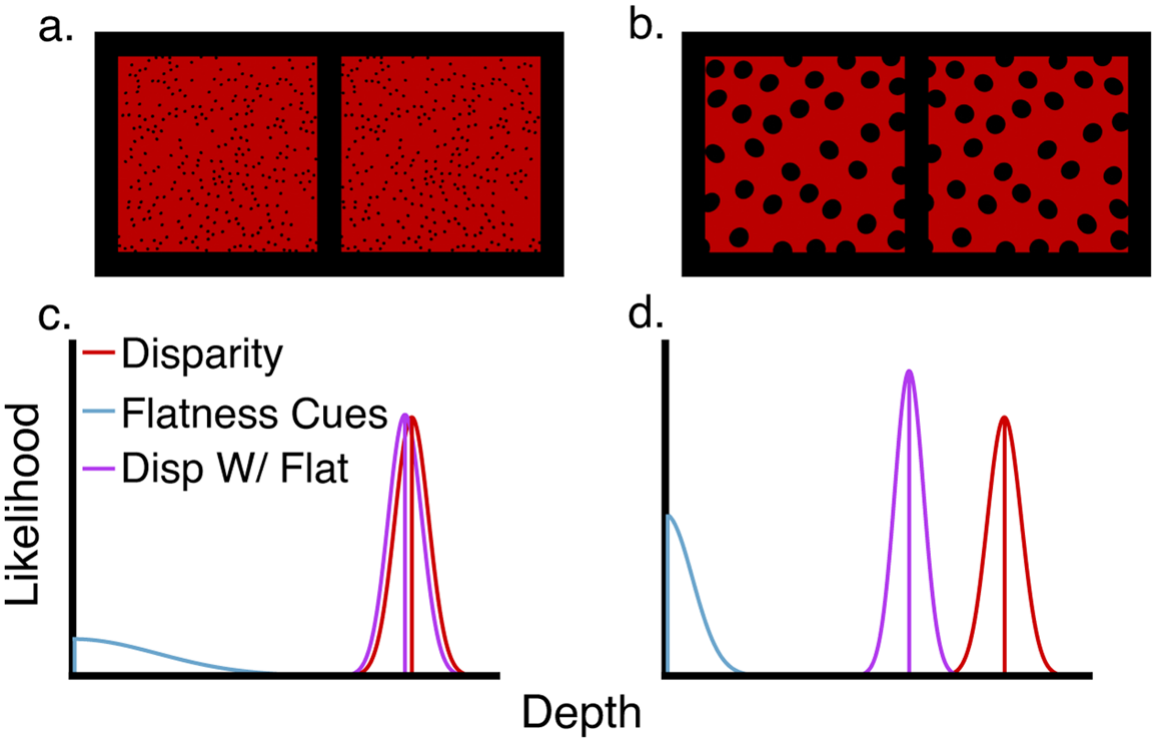
Cross-fusable binocular images of the corrugated surface. According to the MLE model, RDS displays (a) are expected to provide reliable depth estimates only from binocular disparity, given the very low reliability of the texture information (c). The red distribution represents the likelihood for depth from disparity and the cyan distribution the likelihood of flatness cues. The violet distribution shows the optimally combined distribution according to MLE. Note how the center of the distribution is only slightly pulled toward flatness. Unlike RDS displays, large polka dots (b) on the image plane reliably specify a flat frontoparallel surface. This flatness cue therefore produces a sharply peaked likelihood function centered at 0 depth (cyan). In this case the peak of the combined estimate is significantly pulled toward a flatter depth estimate (violet) (d). In contrast, the vector sum model predicts the same depth estimate in both conditions.

Figure 10 illustrates the effects of flatness cues for the MLE model for corrugated surfaces rendered with either a RDS (Figure 10a) or flat textural cues (Figure 10b). Both pairs of images were constructed through back-projecting polka dot texels from a flat surface onto the corrugated surface with respect to the cyclopean eye. Although there are distortions in the individual monocular images, cross-fusing the images will cause the texture to appear as circular polka dots. Despite the texture in both stimuli signaling a flat surface, the enlarged textures of Figure 10b are expected to increase the reliability of the flatness cue due to the more noticeable absence in compression gradient. Figure 10c shows the predictions of the MLE model for the RDS (Figure 10a). As there is potentially some residual texture information from the random-dots, this cue is represented as a zero-mean, large-variance distribution (blue). When combined with the reliable disparity cue (red), it has a negligible influence on the combined-cue estimate (purple). However, texture information is much more reliable for the polka dot stimulus containing a textural flatness cue (Figure 10b). Thus, in Figure 10d, the texture cue is represented as a zero-mean, small-variance distribution (blue). Consequently, when combined with the same reliable disparity cue (red), it will exert a larger influence on the combined cue estimate (purple).

In the next experiment, we compared the predictions of the two models by testing whether intentionally adding flatness cues would reduce the perceived depth of a stimulus. In Experiment 3A, we compared perceived depth under monocular versus binocular viewing of the texture stimulus from Experiment 1. Binocular viewing of a texture stimulus with disparities indicating a frontoparallel surface provides a reliable flatness cue, akin to viewing a picture on a printed page, whereas monocular viewing of the same stimulus provides no such cue from disparities. Under the vector sum model, monocular and binocular viewing of a stimulus are equivalent, as they both have the effect of nullifying the disparity term in the vector sum equation (by setting either *k_d_* = 0 or *z_d_* = 0, respectively). Under the MLE model perceived depth should be greatly reduced under binocular viewing compared with monocular viewing, as disparities are posited to be highly reliable at near viewing distances, such that the disparity weight may exceed the texture weight. In Experiment 3B, we presented stimuli with the opposite relationship: binocular disparities provided non-zero depth information, but they were paired either with a textural flatness cue from a well-defined pattern specifying a frontoparallel surface, or with an uninformative random-dot pattern often used to eliminate pictorial information from disparity stimuli (Figure 10). Here, the predictions are similar. The vector sum model predicts no difference in perceived depth, while the MLE model predicts a measurable difference.

### Experiment 3: Methods

#### Participants

Seven observers participated in Experiment 3A, including two of the authors. Seven additional observers participated in Experiment 3B.

#### Apparatus

In Experiment 3A, the setup was the same as in Experiment 1, except that participants wore only PLATO shutter glasses (Translucent Technologies, Inc., Toronto, ON, Canada), which were used to occlude the vision of the left eye during monocular viewing. Experiment 3B was conducted on a different system but using a similar setup: Alienware A51 with NVIDIA Quadro RTX 4000 GPU, G90fB Graphics Series CRT monitor (resolution 1280 × 1024, refresh rate 60 Hz; ViewSonic, Brea, CA), and Edge RF controlled shutter glasses (Volfoni, Paris, France).

#### Procedure

The stimuli and procedures were similar to Experiment 1 with a few exceptions. In Experiment 3A, the stimuli were defined by texture and shading cues (referred to as texture for simplicity; see Figure 3). However, the same image was presented to the left and right eyes, producing disparities indicating a frontoparallel surface. Monocular and binocular viewing were randomly intermixed within the experiment using the PLATO shutter glasses.

In Experiment 3B, participants judged the depth specified by disparity information in two conditions. The RDS (no-texture) condition was similar to the disparity condition of previous experiments, except that the dots were painted black on a red background square subtending 8° of visual angle (along the diagonal) with an average of 292 visible dots. The dots subtended a visual angle of 0.05^o^. In the flat-texture condition, we created a binocular stimulus that projected circular, 0.55° polka dots on the image screen by back-projecting the frontoparallel texture onto the corrugated surface (see Figures 10a and 10b for example RDS and flat texture with 15 mm of depth). Unlike Experiment 1, we changed the RDS display to have black dots on a red surface so that the only differences between conditions were the size and distribution of the texture elements. According to the MLE model, the reliability of flatness cues is markedly different for these two stimuli. For the RDS condition (Figure 10a), the reliability of the texture information was approximately negligible, as argued by Hillis et al. (2004) when they tested the integration of texture and disparity cues. In contrast, large circular disks randomly positioned on the image (Figure 10b) specify a flat surface in the frontoparallel plane. If flatness cues have any bearing on depth perception as predicted by the MLE model, then we should expect that the flat-texture condition would induce a sizeable flattening of the perceived amplitude of the sinusoidal corrugation. However, the vector sum model predicts no difference between the two conditions.

#### Experiment 3A: Results

**Figure 11.**
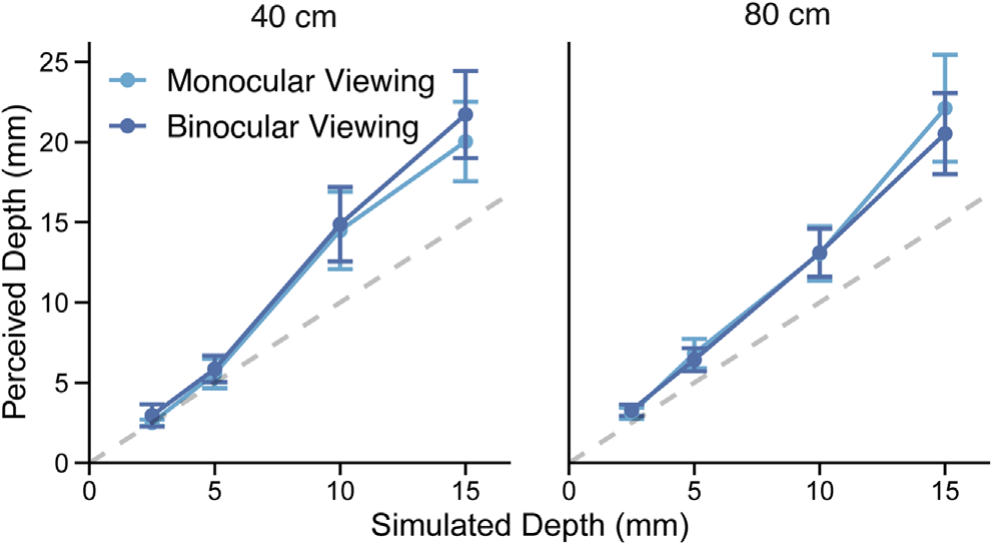
Averaged perceived depth as a function of simulated depth in Experiment 3. Depth information is specified by texture and shading cues. Dark blue circles represent binocular view of the flat picture plane. Light blue squares represent monocular view. Error bars show the *SE* around the average response.

Figure 11 plots the average perceived depth as a function of simulated depth for monocular (light blue) and binocular viewing (dark blue) at the two viewing distances. Repeated-measures ANOVA revealed a significant effect of simulated depth, *F*(1, 6) = 49.94, *p* = 4.0e-4, generalized η^2^ = 0.86. There were no other significant main effects or interactions. It may be noted that the average perceived depth in this experiment was larger than in Experiment 1. This is most likely due to the context of this experiment where trials showing only monocular cues were not interleaved with trials containing multiple cues. It could be the case that in Experiment 1 observers adjusted their criteria so that they reported relatively smaller depth magnitudes for monocular cues. Additionally, individual differences were large (compare the representative subject in Figure 7a to the average in Figure 3), so the increase may be due to sampling differences. What is critical is that binocular viewing did not reduce perceived depth.

To evaluate the support for the vector sum model prediction of no difference between binocular and monocular viewing (i.e., the null hypothesis), we conducted a Bayes factor analysis. A Bayes factor of 0.21 indicated moderate evidence for a model including fixed effects of simulated depth and viewing distance and a random effect for participants, compared with a model including all three effects with an additional fixed effect of viewing condition. This supports the vector sum model prediction that the zero-disparity field specifying the flat picture plane does not influence perceived depth (see also Vishwanath and Hibbard, 2013).

These findings seriously call into question the idea that the pattern of results observed in Experiment 1 (and in previous studies) is due to flatness cues. Moreover, the fact that depth perception is unaltered when viewing a pictorial stimulus with one or two eyes is successfully accounted for by the vector sum model.

#### Experiment 3B: Results

**Figure 12.**
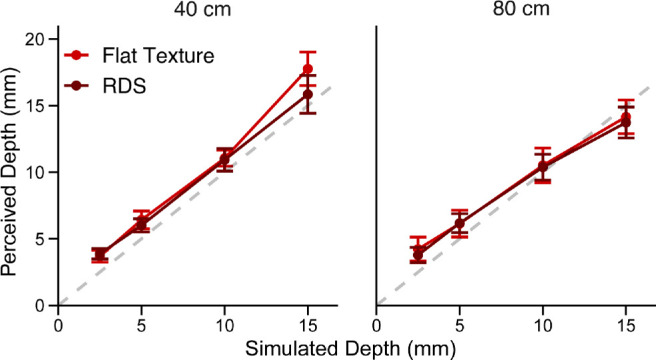
Averaged perceived depth as function of depth from binocular disparities in Experiment 3b. Bright red indicates the RDS condition. Dark red indicates the viewing condition where polka dot texture specified a flat frontoparallel surface. Error bars show the *SE* around the average response.

Figure 12 plots the perceived depth estimates in the flat-texture (bright red) and RDS (dark red) conditions. Repeated-measures ANOVA revealed a significant main effect of simulated depth, *F*(1, 6) = 216.78, *p* = 6.2e-6, generalized η^2^ = 0.92, and a significant interaction between simulated depth and fixation distance, *F*(1, 6) = 8.28, *p* = 0.028, generalized η^2^ = 0.15. To evaluate the support for the vector sum model prediction of no difference between the flat-texture and RDS, we again conducted a Bayes factor analysis. A Bayes factor of 0.42 indicated anecdotal evidence for a model including fixed effects of simulated depth and viewing distance and a random effect for participants, compared with a model including all three effects with an additional fixed effect of viewing condition. Together, the results of these experiments support the vector sum model prediction that there is no difference between setting the depth of a cue to zero or eliminating the cue altogether.

## General discussion

The results of these three experiments challenge three fundamental assumptions of previous models of 3D cue integration:

• *Veridicality*—Independent visual modules compute the veridical metric structure of 3D objects from retinal projections.
• *Probabilistic inference*—The output of each module is a likelihood distribution of all possible 3D structures that may have generated a given retinal image. The width of these distributions is a measure of the perceptual estimation noise from each individual cue. In other words, each module has explicit access to information about the reliability of a given visual input.
• *Statistically optimal combination*—3D cue estimates are optimally combined by computing the joint probability distribution from the independent probability distributions of each individual cue. The perceptual estimate corresponds to the 3D structure that maximizes this joint probability distribution. Moreover, because the joint probability distribution has a smaller variance than that of each individual cue the combined estimate is also more reliable. In the case of the linear MLE model, a simple heuristic can achieve a statistically optimal combination, where single-cue estimates are combined through a weighted average where the weights are inversely proportional to the variance of the noise of single-cue estimates.

The veridicality assumption is clearly contradicted by the results of the first experiment, where participants judged the amplitude of a surface with a sinusoidal probe. Following a classic cue combination paradigm, we studied these depth judgments with disparity-only, texture-only, and combined-cue stimuli. In most of these conditions, the perceptual slopes relating simulated depth to perceived depth differ from a slope of one and they significantly differ from each other. Moreover, the biases observed in single-cue conditions do not diminish when cues are combined.

The probabilistic inference assumption is challenged by the results of the second experiment where we showed that JNDs measured in a depth discrimination task were inversely proportional to the slope of the perceptual function independently measured in the first experiment. Because the perceptual slope is sufficient to predict depth discrimination, it presents a valid alternative interpretation of JNDs from the one postulated by the MLE models. Moreover, the IC theory explanation is more parsimonious because it does not assume mechanisms that have access to explicit measures of the reliability of the visual input.

The statistically optimal combination assumption is contradicted by the results of all three experiments. In the first experiment, we found that the perceptual slope in the combined-cue condition was larger than the perceptual slope in the single-cue conditions, a result incompatible with the prediction of weighted cue combination of the linear MLE model. In the second experiment, we showed that the smaller JND in the combined-cue condition relative to the single-cue conditions could be explained by the larger perceptual slope. In the third experiment, we showed that adding a reliable flatness cue to a 3D stimulus did not produce a significant reduction in depth magnitude. This finding contradicts the weighted-cue combination rule of the MLE model, because adding a reliable flatness cue should produce a weighted average that is biased toward flatness. These results should especially be expected when a flat disparity field is added to a texture-specified 3D surface because at close distances disparity information is highly reliable. Instead, we observed no difference in perceived depth magnitudes when the picture of a texture stimulus was seen monocularly or binocularly. This finding also contradicts a possible MLE interpretation of the results of the first experiment. According to this interpretation, the larger slope of the combined-cue condition relative to the single-cue conditions may be attributed to the influence of spurious flatness cues affecting stimuli rendered on flat CRT displays. The larger slope in the combined-cue condition, then, is due to these flatness cues influencing single-cue estimates to a greater extent than combined-cue estimates, as the former are less reliable than the latter. If this explanation is correct and spurious flatness cues such as the blurring gradient noticeably influence depth estimates, then we should expect an even larger effect when we introduce highly reliable flatness cues such as a flat disparity field. But, this is not what we found. In contrast to the observed discordance between the empirical data and the predictions of the MLE model, these findings can be accounted for by the IC theory of cue integration. These results therefore have significant theoretical implications because the IC theory rejects the fundamental hypotheses on which the MLE theory and the Bayesian approach in general stand.

### Linear mapping versus veridical perception

The first important departure of the IC theory from previous theories is the rejection of metric accuracy as the normative goal of 3D processing. For the IC theory, mechanisms performing independent computations on the visual input derive 3D estimates that are *linearly* related to distal properties but are in general inaccurate. The slope of these linear functions, which we term *cue strength*, depends on the quality of the visual input. For example, a regular pattern of texture elements on a distal surface such as polka dots will produce a larger texture signal than sparse texture elements. Therefore, a texture module will in the first case exhibit a steeper input–output perceptual function than in the second case. Similarly, a disparity module will respond with a steeper perceptual function to the depth of objects at closer distances than at further distances. The results of Experiment 1 show indeed that depth judgments are not veridical and depend on the viewing conditions. It can be observed that the perceptual slope in the disparity condition is shallower at a viewing distance of 80 cm than at 40 cm. At the smaller distance, depth from disparity is overestimated, and it is larger in magnitude than depth from texture. However, at the larger distance these estimates are almost the same.

### Deterministic versus probabilistic mapping

The second fundamental difference between the IC theory and MLE model is that the output of visual modules is *deterministic* and does not carry any information about the reliability of the input. Consider again a texture gradient projected by sparse surface texture elements. For the MLE account this is an unreliable image signal that produces a noisy output. In other words, each time similar (i.e., equally unreliable) stimuli are viewed, the texture module will provide a different depth estimate. However, according to the veridicality assumption, the average estimate arising from multiple measurements will be unbiased. In contrast, the IC theory will derive similar depth estimates albeit much smaller than the distal depth magnitude. What the MLE approach considers unreliable cues are weak cues for the IC theory because a change in distal depth elicits a small change in the module output.

The deterministic nature of the mapping between distal and derived depth postulated by the IC theory requires an adequate re-interpretation of perceptual variability in depth estimation tasks. The most radical re-interpretation of variability measurements is with respect to the JNDs observed in the depth discrimination tasks. The MLE model considers the JND as a proxy measure of the standard deviation of the noise underlying perceptual estimates of depth. However, according to the IC theory, the noise influencing discriminability does not stem from variability of depth estimates but, instead, from task-related processes. In the specific case of a 2IFC task, memory retention and retrieval of the stimulus presented in the first interval is subject to “smearing” (Rademaker, Park, Sack, & Tong, 2018), thus affecting the following comparison with the stimulus presented in the second interval. To overcome this memory-related noise, the perceived depth magnitude of the two stimuli must differ by some minimum amount. Although the perceived depth difference necessary for a reliable discrimination is fixed, the *simulated* depth difference required to yield this perceived depth difference depends on the cue strength. Therefore, the JND, defined as the simulated depth difference necessary for a reliable discrimination, is inversely proportional to the cue strength.

It is important to note that noise due to memory retention/retrieval is especially relevant for the commonly adopted 2IFC task, but other noise sources affect different kinds of tasks. Consider, for example, comparing the size of a 2D probe to a simultaneously present 3D stimulus, as in Experiment 1. This task involves difficult cognitive operations, such as mental rotation, which generate uncertainty. However, the fundamental assumption of the IC theory is that any kind of task-related noise is predicted to be independent from the cue or combination of cues defining a 3D shape. Indeed, this can be clearly seen in Figure 5, where the standard deviation of the probe judgments does not depend on the cue type but on the perceived depth magnitude, as predicted by the Weber law applied to perceived quantities.

This novel interpretation of the JND is sufficient to predict the data of the second experiment since the observed JND is proportional to the inverse of the cue strength. Moreover, as we will discuss shortly, the vector sum model of the IC theory and the alternative interpretation of discrimination thresholds yield the same predictions as the MLE model for the JND of combined-cue stimuli.

### Vector sum versus probabilistic inference

Within the IC framework, independent depth modules have a deterministic input–output mapping; that is, the same type of visual input elicits the same output. However, this does not mean that the output of a 3D module is not subject to undesired fluctuations. The important distinction between the MLE theory and the IC theory resides in the nature of these fluctuations. For the MLE model, the inferential process interpreting an unreliable visual input will produce large variations in the output estimates because even slight changes in the input will result in large perturbations of the associated likelihood function (Ernst & Banks, 2002; Held, Cooper, & Banks, 2012; Hillis et al., 2004; Knill 1998a; Knill 1998b; Knill, 2003; Knill & Saunders, 2003). Due to inferential process, trial-by-trial error emerges in the depth estimate. It therefore makes intuitive sense that the linear MLE model combines visual estimates with weights that are inversely related to the variance of the output noise. Note, however, that the weights must be estimated at each single instance and therefore visual modules must carry information about the reliability of a given visual input.

For the IC theory, fluctuations of a module output are caused by changes in the quality of the visual input. For example, the same distal structure will yield 3D estimates of different magnitudes depending on the nuisance variables characterizing different viewing conditions where the object has different surface properties (e.g., texture and reflectance functions), is seen from different viewing distances, is subject to different illuminants, and so on. Therefore, the IC theory postulates that the goal of the visual system is to attenuate as much as possible the influence of the nuisance variables to approach depth constancy in the face of varying viewing conditions. It can be shown that the vector sum of the appropriately scaled module outputs minimizes the undesired influence of scene parameters while maximizing the sensitivity to distal depth changes (Appendix 1). This simple rule of cue combination yields specific predictions regarding both (1) the magnitude of depth judgments and (2) the discrimination thresholds of combined-cue stimuli. The first prediction is that the perceived magnitude of combined-cue stimuli is equal to the vector sum of the perceived magnitude of single-cue stimuli. Specifically, the cue strength (i.e., perceptual slope) of the combined-cue stimuli is the vector sum of the strengths of the single-cue stimuli.

This prediction is confirmed by the results of the first experiment. The second prediction follows from the first. Because, according to the IC theory, the JND is inversely proportional to the perceptual slope, it follows that the JND of the combined-cue stimuli is smaller than the JND of the single-cue stimuli (Appendix 3). Notably, the predicted reduction in magnitude of the JND for the combined-cue stimuli is identical to that of the MLE model. The algebraic equivalence of the vector sum and MLE predictions of the JND expected from cue combination validates the IC theory because it can account for many empirical findings that use depth discrimination to support the MLE predictions (Hillis et al., 2004; Knill & Saunders, 2003). Finally, the vector sum combination rule also predicts the results of the third experiment. When a flatness cue is present in a display, its contribution to the vector sum is equivalent to that of an absent cue. For example, when looking at a picture with only one eye, no disparity information is present, whereas when looking at the same picture with two eyes the disparity field specifies zero depth. In both cases the contribution of the disparity term is nil.

## Conclusions

In this study, we tested the predictions of a new theory of depth cue integration termed the intrinsic constraint (IC) theory. This theory postulates the existence of independent modules relating distal 3D properties to perceived 3D properties through deterministic functions that are, in optimal conditions, linear. The slopes of these functions depend on scene parameters specific to the viewing conditions. In ideal viewing conditions depth modules are highly sensitive to distal changes in 3D properties, as, for example, when the surface properties of an object produces a larger texture gradient. However, in viewing conditions where 3D information from a specific cue provides poor information, as for an object that only has very sparse texture elements on its surface, the response of the depth module will be shallow. The IC theory combines individual estimates through a vector sum that maximizes the response to changes in distal 3D properties while minimizing the module-output fluctuations due to varying scene parameters.

We tested this model in three experiments targeting different aspects of 3D shape judgments. First, we confirmed the prediction that increasing the number of cues specifying a 3D surface will increase the perceived depth of that surface, a hypothesis emerging from the vector sum model. This result has been recently found in other studies using grasping to test depth perception in both virtual reality environments and with real objects (Campagnoli & Domini, 2019; Campagnoli, Hung, & Domini, 2022). Although Bayesian models can account for the phenomenon predicted by the vector sum model, the IC theory has the significant advantage of achieving the same predictions without the need for further ad hoc assumptions such as flatness cues or a flatness prior (Di Luca, Domini, & Caudek, 2010; Domini and Caudek, 2003; Domini and Caudek, 2009; Domini and Caudek, 2010; Domini and Caudek, 2011; Domini and Caudek, 2013; Domini et al., 2006; Domini, Shah, & Caudek, 2011). This advantage is not confined to the case of depth cue integration, but it applies to other common visual experiences such as picture perception. In this case, too, neither flatness cues nor a flatness prior would appear to be able to explain the empirical data. Second, we tested the ability of the IC theory to predict the JND of a combined-cue stimulus from the JNDs of single-cue stimuli. Notably, the IC theory makes predictions that are formally identical to those of Bayesian models, therefore accounting for a number of previous investigations that leverage JND data as the strongest source of evidence in support of Bayesian cue combination. However, the JND for the IC theory is determined by the slope of the perceptual function and not by the noise of depth estimates.

In summary, the IC theory seems to be a better candidate for explaining 3D cue integration experiments because (1) it can predict previous data in support of Bayesian models, (2) it predicts new results that are incompatible with previous models, and (3) it is more parsimonious, as it does not postulate veridical perception or require estimates of cue reliability that are necessary for the functioning of Bayesian models.

## Footnotes

1 Within this framework, a type of visual information traditionally defined as a single, monolithic cue may in fact be better understood as multiple cues, so long as they are affected by independent nuisance variables. Indeed, by this definition there are several distinct types of texture cues that are traditionally treated as a single cue (Chen & Saunders, 2020; Todd & Thaler, 2010; Todd et al., 2007).

## Appendix Appendix 1: The vector sum equation maximizes the signal-to-noise-ratio

For simplicity, consider only two signals: *s*_1_ = λ_1_*z* and *s*_2_ = λ_2_*z*, where λ_*i*_ are unknown multipliers depending on nuisance variables and *z* is the magnitude of the 3D property. These signals are the visual systems encoding of the 3D information from independent cues (e.g., texture, disparity). We seek an estimate ${\hat{z}}_{C}=f\left(s_{1},s_{2}\right)$ that is (1) proportional to *z* and (2) most sensitive to 3D information and least sensitive to random fluctuations ε_*i*_ of λ_*i*_. If λ_*i*0_ is the unperturbed value of λ_*i*_, then λ_*i*_ = λ_*i*0_ +  ε_*i*_ and *s*_*i*0_ = λ_*i*0_*z*. We assume that small random perturbations, ε_*i*_, resulting from changes in viewing conditions can be modeled as Gaussian distributions with zero mean and standard deviations σ_*i*_. Taking the derivative of $\frac{df(s_{1},\;s_{2})\;}{dz}=\frac{df\;}{ds_{1}}\left(\lambda_{10}+\;{ɛ}_{1}\right)+\frac{df\;}{ds_{2}}\left(\lambda_{20}+\;{ɛ}_{2}\right),$ where $\frac{df\;}{ds_{i}}$ are calculated at *s*_*i*0_, we observe a signal term *S* = *f*_1_λ_10_ + *f*_2_λ_20_ (where $f_{i}=\frac{df\;}{ds_{i}}$) and a noise term E = *f*_1_ε_1_ + *f*_2_ε_2_ having standard deviation $\sigma_{E}=\sqrt{{f_{1}}^{2}\sigma_{1}^{2}+{f_{2}}^{2}\sigma_{2}^{2}}$. If we minimize the noise-to-signal ratio $NSR=\frac{\sigma_{E}\;}{S}$ with respect to *f_i_* (by solving for *f_i_* the equation $\frac{dSNR\;}{df_{i}}=0$, where *SNR* is the signal-to-noise ratio) we find that the first derivatives of the function are $\frac{df\;}{ds_{i}}\propto \frac{\lambda_{i0}\;}{\sigma_{i}^{2}}$. It can be shown that the derivatives $\frac{d{\hat{z}}_{C}\;}{ds_{i}}\;$of the equation ${\hat{z}}_{C}=\beta \sqrt{{\left(\frac{s_{1}\;}{\sigma_{1}}\right)}^{2}+{\left(\frac{s_{2}\;}{\sigma_{2}}\right)}^{2}}$ (calculated at *s*_*i*0_) meet this requirement. By substituting $k_{i}=\beta \frac{\lambda_{i}}{\sigma_{i}}$ we obtain the vector sum equation$\;{\hat{z}}_{C}=\sqrt{{\left(k_{1}z\right)}^{2}+{\left(k_{2}z\right)}^{2}}$ (easily generalizable to *n* signals).

## Appendix 2: The IC theory predicts the same linear combination rule as the Bayesian models in matching tasks

Hillis et al. (2004) predicted the outcome of a task where the perceived slant of a non-conflict stimulus, *S_NC_* = *S_B_* + δ, is matched to that of a conflict stimulus, *S_C_*, where *S_B_* is an arbitrarily defined base slant and δ is the change in slant needed for a perceptual match $E\left({\hat{S}}_{C}\right)=E\left({\hat{S}}_{NC}\right)$. For the conflict stimulus, the disparity slant,*S_D_*, differs from a texture-specified slant, *S_t_*, by Δ: *S_t_* = *S_B_* and *S_d_* = *S_B_* + Δ. Optimal cue combination predicts that$\;E\left({\hat{S}}_{C}\right)=w_{d}\left(S_{B}+\Delta \right)+\left(1-w_{D}\right)S_{B}=\;S_{B}+\;w_{d}\Delta$, where$\;w_{d}=\frac{\frac{1}{\sigma_{d}^{2}}}{\frac{1}{\sigma_{d}^{2}}+\frac{1}{\sigma_{t}^{2}}}$. A matching $E\left({\hat{S}}_{C}\right)=E\left({\hat{S}}_{NC}\right)$ is obtained when $w_{d}=\frac{\delta }{\Delta }$, as $E\left({\hat{S}}_{NC}\right)=S_{B}+\delta$. By using JNDs as proxies for standard deviations the weight can be accurately predicted: $w_{d}=\frac{\frac{1}{J_{d}^{2}}}{\frac{1}{J_{d}^{2}}+\frac{1}{J_{t}^{2}}}$. The IC theory makes identical predictions. For a small conflict Δ, we can approximate the vector sum equation through the Taylor expansion at the base slant *S_B_*: ${\hat{S}}_{C}=\sqrt{{k_{t}}^{2}{S_{B}}^{2}+{k_{d}}^{2}{\left(S_{B}+\Delta \right)}^{2}}\approx S_{B}\sqrt{{k_{t}}^{2}+{k_{d}}^{2}}+\frac{{k_{d}}^{2}}{\sqrt{{k_{t}}^{2}+{k_{d}}^{2}}}\Delta$. Because ${\hat{S}}_{NC}$ = $\left(S_{B}+\delta \right)\sqrt{{k_{t}}^{2}+{k_{d}}^{2}}$=$S_{B}\sqrt{{k_{t}}^{2}+{k_{d}}^{2}}+\delta \sqrt{{k_{t}}^{2}+{k_{d}}^{2}}$, a match, ${\hat{S}}_{NC}={\hat{S}}_{C}$, is obtained when $\frac{{k_{d}}^{2}}{\sqrt{{k_{t}}^{2}+{k_{d}}^{2}}}\Delta =\delta \sqrt{{k_{t}}^{2}+{k_{d}}^{2}}$, from which $\frac{{k_{d}}^{2}}{{k_{t}}^{2}+{k_{d}}^{2}}=\frac{\delta }{\Delta }$. Note that, because for the IC theory $J_{i}=\frac{\sigma_{N}}{k_{i}}$ (see Introduction to Experiment 2), then $\frac{{k_{d}}^{2}}{{k_{t}}^{2}+{k_{d}}^{2}}=w_{d}$, which matches the predictions of Hillis et al. (2004).

## Appendix 3: The vector sum model predicts the same JND of combined stimuli as that predicted by linear MLE combination

The MLE model predicts that, when two cues with independent Gaussian noise of standard deviation $\sigma_{i}^{2}$ are combined through a weighted average with weights inversely proportional to the variance of each cue, then the combined (inverse) variance is $\frac{1}{\sigma_{c}^{2}}=\frac{1}{\sigma_{1}^{2}}+\frac{1}{\sigma_{2}^{2}}$. If JNDs are proxies for the standard deviations, then $\frac{1}{J_{c}^{2}}=\frac{1}{J_{1}^{2}}+\frac{1}{J_{2}^{2}}$. For the IC theory, JNDs depend on the task noise σ_*N*_ and gain *k_i_*: $J_{1}=\frac{\sigma_{N}}{k_{1}}$ and $J_{2}=\frac{\sigma_{N}}{k_{2}}$. Because from the vector sum equation the gain of the combined stimulus is $k_{c}=\sqrt{{k_{1}}^{2}+{k_{2}}^{2}}$, the JND of the combined stimulus is $J_{c}=\frac{\sigma_{N}}{k_{c}}=\frac{\sigma_{N}}{\sqrt{{k_{1}}^{2}+{k_{2}}^{2}}}$. By substituting $k_{i}=\frac{\sigma_{N}}{J_{i}}$ in this equation we obtain $J_{c}=\frac{1}{\sqrt{\frac{1}{J_{1}^{2}}+\frac{1}{J_{2}^{2}}}}$, which is identical to the MLE prediction.
